# Supplementary material for: The essential genome of the crenarchaeal model Sulfolobus islandicus
Source: Nat Commun. 2018 Nov 21;9:4908. doi: 10.1038/s41467-018-07379-4 (PMC6249222; doi:10.1038/s41467-018-07379-4)
Supplement: Supplementary file 1 — Supplementary Information [file 41467_2018_7379_MOESM1_ESM.pdf]

The essential genome of the crenarchaeal model *Sulfolobus islandicus*

Changyi Zhang[1, 2#], Alex P. R. Phillips[1, 2#], Rebecca L. Wipfler [1], Gary J. Olsen[1, 2] and Rachel J. Whitaker[1, 2*]

[1.] Carl R. Woese Institute for Genomic Biology, University of Illinois at Urbana-Champaign, Urbana, Illinois, USA

[2.] Department of Microbiology, University of Illinois at Urbana-Champaign, Urbana, Illinois, USA

[#] C.Z. and A.P.R.P contributed equally to this work

[*]Correspondence: Rachel J. Whitaker. E-mail: rwhitaker@life.illinois.edu

Supplementary Notes 1-5

Here we provide additional descriptions and discussion of essential genes in several arCOG functional categories from the perspective of gene function, and highlight a few non-essential genes that possibly play critical roles in *Sulfolobus* species.

1. DNA replication, repair, and recombination

Tn-seq analysis allowed us to identify 23 essential genes involved in DNA replication, repair, and recombination (arCOG functional category [L]) in *S. islandicus*, among which 14 genes encode core components of archaeal DNA replication machinery. The MCM (mini-chromosome maintenance complex), one of the indispensable ancillary complexes during DNA replication in Archaea and Eukaryotes, is the replicative helicase for DNA unwinding, performing the function of DnaB in Bacteria. In contrast to the two hyperthermophilic euryarchaea *Methanococcus maripaludis* and *Thermococcus kodakarensis*, which possess multiple *mcm* genes with only one copy required[1-3], *Sulfolobus* species contain only one MCM, forming a homohexameric architecture[4]. Given the indispensable roles of MCM for DNA unwinding during DNA replication, the existence of the sole *mcm* gene in *S. islandicus* explains why the inactivation (via transposon insertion or gene knockout strategy) of *mcm* is lethal. Notably, the essentiality of the single *mcm* gene was also demonstrated in a halophilic archaeon, *Halobacterium* sp. NRC-1[5]. Another two genes involved in the initiation of DNA replication encode GINS homologs Gins23 and Gins15, both of which are essential in *S. islandicus* M.16.4. Additionally, the archaeal ortholog of Cdc45, which was shown to form a stable complex with GINS to stimulate MCM helicase activity in *S. solfataricus*[6], was essential in our study. The essentiality of *gins*, *mcm*, and *cdc45* further supports a view demonstrating the formation of CMG complex is required for DNA replication in *Sulfolobus*[6]. Intriguingly, recent genetic studies in the hyperthermophilic euryarchaeon *T. kodakarensis* revealed the Cdc45/RecJ-like protein encoding gene *gan* was not essential for cell viability[7,8], indicating the function of CMG complex presumably diverged in archaea.

PCNA (Proliferating cell nuclear antigen) belonging to the family of DNA sliding clamps is structurally and functionally conserved[9,10]. Unlike Euryarchaeota, which generally contain one PCNA gene (with the exception of *T. kodakarensis* in which two PCNA homologs were found[11,12]), Crenarchaeota possess three distinct PCNA subunits. All three subunits were essential in *S. islandicus* M.16.4 inferred by Tn-seq data, consistent with a previous genetic analysis in another *S. islandicus* strain Rey15A[13]. These results explain why three PCNA subunits formed a heterotrimer rather than distinct homotrimers to act as the sliding clamp during DNA replication in other *Sulfolobus* species[14]. The two subunits of replication factor C

(RFC_S and RFC_L), acting as the loader of the DNA sliding clamp, are found in all three domains and are essential in *S. islandicus* M.16.4 as well as the euryarchaeon *M. maripaludis*[1].

One of the unique features of Archaea is that they exclusively encode both bacterial-type (DnaG) and eukaryotic-type primase, with the later type consisting of a small subunit (catalytic subunit, PriS) and a large subunit (noncatalytic subunit, PriL). The function of DnaG and heterodimer PriLS have been previously biochemically characterized in *S. solfataricus*[15,16]. Strikingly, a novel primase PriX was recently identified and shown to significantly promote the primer synthesis *in vitro* by forming a heterotrimer with PriSL in *S. solfataricus*[17]. Here we revealed *dnaG* in *S. islandicus* (*M164_2048*) was essential, in contrast to the non-essentiality of *dnaG* in the euryarchaea *M. Maripaludis* and *Haloferax volcanii*[1,16]. Tn-seq data showed *priS* (*M164_1162*) was essential whereas *priL* (*M164_1568*) and *priX* (*M164_1652*) were classified as "unassigned". Moreover, we were unable to obtain knockouts for *priL* and *priX* even if we prolonged the incubation of transformation plates for 20 days, indicating they are required for cell survival in *S. islandicus* M.16.4. There are another two "unassigned" genes related to DNA maturation: *lig*, encoding an ATP-dependent DNA ligase[18], and *rnhII*, encoding a ribonuclease HII that was shown *in vitro* to exhibit the cleavage activity of RNA in hybrid RNA/DNA substrates in *S. tokodaii*[19]. All attempts to delete *lig* or *rnhII* in *S. islandicus* M.16.4 were unsuccessful, confirming that they are required for the primer removal during the maturation of Okazaki fragments in *Sulfolobus* DNA replication. Notably, this result argues against genetic studies performed in the hyperthermophilic euryarchaeon *T. kodakarensis* in which the function of RNase HII can be replaced by the Fen1 or GAN (GINS-associated nuclease)[7].

Sulfolobus species encode three B-family DNA polymerases and one Y-family DNA polymerase[20]. However, only *dpoB1* (*M164_1573*) is essential whereas *dpoB2* (*M164_0814*), *dpoB3* (*M164_2047*), and *dpo4* (*M164_0255*) are classified as non-essential via Tn-seq analysis. To validate these results, direct gene disruptions were attempted by using the *argD* marker cassette to replace the *dpoB1*, *dpoB2*, *dpoB3*, and *dpo4* in the chromosome. Consequently, individual disruption mutants of *dpoB2*, *dpoB3*, and *dpo4* could be successfully obtained (Supplementary Fig. 3b); however, disruption of *dpoB1* failed after repeated attempts. These studies suggest DpoB1 is an authentic replicative DNA polymerase *in vivo* for *Sulfolobus*, although *in vitro* studies showed DpoB2 and DpoB3 possessed very low DNA polymerase and 3′ to 5′ exonuclease activities[21], consistent with phylogenetic analyses suggesting the B-family DNA polymerases evolved by gene duplication events in Crenarchaeota[22,23]. *S. islandicus* M.16.4 encodes PolB1-binding proteins PBP1 (M164_1996;

arCOG functional category [S]) and PBP2 (M164_1545; arCOG functional category [K]), the orthologues of which have been recently identified to form a heterotrimeric DNA polymerase holoenzyme together with DpoB1 in a related species *S. solfataricus*[24]. Both Tn-seq and gene knockout analyses showed that *pbp2* was essential whereas *pbp1* was not (Supplementary Fig. 3b), indicating the formation of heterotrimeric DNA polymerase holoenzyme was not necessary *in vivo*, at least for the maintenance of cell growth in *S. islandicus*. Remarkably, with the exception of the two newly isolated thermophilic ammonia-oxidizing thaumarchaea *Candidatus Nitrosocaldus cavascurensis* and *Candidatus Nitrosocaldus islandicus*[25,26], which lack archaeal specific D-family DNA polymerase, both B- and D-family DNA polymerases are present in all other members of Thaumarchaeota lineage as well as Euryarchaeota, Korarchaeota, Aigarchaeota, and Nanoarchaeota lineages. Moreover, genetic studies in both *T. kodakarensis* and *M. maripaludis* revealed Pol D was possibly the major DNA replicative polymerase because *dpoB* was non-essential *in vivo*[1,27]. While Crenarchaeota and Eukaryota both use B-family polymerases to replicate their genomes, which can be interpreted as evidence for their shared ancestry, a recent review of the archaeal tree of life suggested that loss of Pol D instead occurred twice independently in the two lineages[28]. Other essential genes in arCOG functional category [L] included *rpa* (*M164_0180*), *top6A* (*M164_1238*), *top6B* (*M164_1239*), and *cren7* (*M164_1232*) encoding a highly conserved chromatin protein in Crenarchaeota[29]. The 5’ flap endonuclease (M164_1965, FEN1) that strongly interacts with PCNA2[14] is essential, whereas in Euryarchaeota it was previously shown that *fen1* could be disrupted or deleted[1,7].

DNA damage repair in *Sulfolobus* species remained largely elusive and most of the predicted candidates related to DNA repair are non-essential (Supplementary Table 3). In particular, the canonical DNA mismatch repair pathway has not been found in *Sulfolobus* species so far. Recently, Ishino *et al*. reported that a mismatch-specific endonuclease (EndoMS) in *T. kodakarensis* could specifically cleave dsDNA substrates with mismatched bases incorporated[30]. The EndoMS was found to be present in some bacteria particularly in Actinobacteria, and distributed in archaeal members belonging to the TACK superphylum, Euryarchaeota, and ASGARD phylum[31,32]. Here we showed that the *S. islandicus* EndoMS homolog (M164_0025; annotated as NucS), existing in all sequenced *Sulfolobus* species, was non-essential by both Tn-seq and gene knockout experiments (Supplementary Fig. 3b). Investigation of the spontaneous mutation rates and mutation spectra in wild-type and *endoMS* mutant strains with a forward mutation assay will be required to identify whether the *Sulfolobus* EndoMS homolog plays a functional role in the mutation avoidance, as recently reported in *Mycobacterium tuberculosis*[32] and *Corynebacterium glutamicum*[33]. The *udg4* (*M164_0085*), encoding uracil-DNA glycosylase, was classified as “unassigned” by Tn-seq assay. We

speculate the *udg4* mutant colonies generated by the insertion of transposon were not successfully captured on plates within 10 days of incubation, which was supported by the observation of the *udg4* knockout mutant exhibiting a greatly reduced cell viability in comparison to the wild type strain (Supplementary Fig. 2b). Four genes *nurA* (*M164_0062*), *rad50* (*M164_0063*), *mre11* (*M164_0064*), and *herA* (*M164_0065*), relevant to double-strand DNA break repair (DSB), were essential in *S. islandicus* M.16.4 as revealed by Tn-seq data, consistent with previous genetic analyses in *S. islandicus* Rey15A and *T. kodakarensis*[34,35]. The gene that encodes RadA (*M164_1897*), the archaeal ortholog of RecA/Rad51 family recombinase, was essential in our study. Genetic analysis of *radA* in *S. islandicus* Rey15A and *T. kodakarensis* showed that mutation of this gene was lethal[34,35], whereas the *H. volcanii* strain lacking *radA* was viable but defective in homologous recombination[36]. More interestingly, it has been shown recently that *radA* was required for the cell survival of *H. volcanii* that lacked all four replication origins[37]. In contrast to hyperthermophilic archaea, *radA*, *rad50*, and *mre11* individual deletion mutants have been successfully generated in mesophilic archaea *Halobacterium salinarum* or *H. volcanii*[36,38-40]. The apparent essentiality of HR-related genes in hyperthermophilic archaea suggested that they presumably harbor a unique mechanism, which is different from that of mesophilic archaea, in order to adapt to their harsh environments, particularly elevated temperatures[41].

2. Transcription

Tn-seq analysis predicted that 11 among 13 RNAP subunits in *S. islandicus* M.16.4 were essential. The Archaea-specific Rpo13 (M164_1754), a RNAP–DNA stabilization factor[42], was identified as non-essential by Tn-seq analysis and successful construction of a *rpo13* disruption mutant (Supplementary Fig. 3b). The RNAP subunit Rpo8 (M164_1872) found in many eukaryotes and highly conserved in Crenarchaeota and Korarchaeota[43], was categorized as "unassigned" (log_2FC=-3.77 and EI=2). Further genetic analysis revealed the disruption of *rpo8* was not lethal (Supplementary Fig. 3b); however, growth of the *rpo8* disruption mutant was significantly impaired compared with that of the wild type strain (Supplementary Fig. 2c). The dispensability of Rpo8 or Rpo13 suggests a complete RNAP, consisting of 13 subunits[42,44], is not required to maintain cell survival *in vivo* for *Sulfolobus*, at least in *S. islandicus* M.16.4.

Like that of eukaryotes, transcription initiation in Archaea required the TATA-box binding protein (TBP) and transcription factor B (TFB) bound to DNA for promoter-dependent transcription. Both Tn-seq and knockout analyses confirmed that the TBP-encoding gene (*M164_1259*) was essential whereas the TBP-interacting protein TIP49[45] encoded by *M164_0257* was non-essential in *S. islandicus* (Supplementary Table 3 and Supplementary Fig. 3b). Three TFB paralogues, encoded by *tfb1* (*M164_1706*), *tfb2* (*M164_1265*), and *tfb3*

(*M164_1868*) respectively, were found in the genome of *S. islandicus* M.16.4. The genes *tfb1* and *tfb2* were essential whereas *tfb3* was not, which were confirmed by both Tn-seq and knockout analyses (Supplementary Fig. 3b). In agreement with our discoveries, similar results regarding the essentiality/non-essentiality of these three TFB paralogs have also been observed in *S. acidocaldarius* via gene disruption analyses[46]. The TFB1 has been identified *in vitro* as one of three indispensable factors to direct accurate transcription in *S. shibatae*[47], whereas the TFB2 was proposed to be involved in the regulation of cell cycle in *S. acdocaldarius*[48]. Additionally, *tfb3* was found to be highly transcribed after UV treatment in *S. solfataricus* and *S. acdocaldarius*[49,50]. Strikingly, it has been shown recently that *tfb3* could regulate the expression of genes involved in cellular aggregation and DNA transfer when the cells were subjected to NQO (4-nitroquinoline 1-oxide) or UV- induced DNA damage[51,52].

An additional transcription factor IIE-α (renamed as TFEα, M164_1881) was annotated in the genome of *S. islandicus* M.16.4 and was revealed to be essential by Tn-seq and gene knockout analyses. Notably, the archaeal counterpart of TFIIEβ (renamed as TFEβ) has been functionally characterized in *S. solfataricus* recently[53], and the TFEβ homolog in *S. islandicus* (M164_1266) was shown to be essential in our study, consistent with the genetic analysis performed in *S. acidocaldarius*[53]. Four genes *spt4* (*M164_1736*), *nusG* (*M164_1807*), *nusA* (*M164_1922*), and *nusA*-like (*M164_1973*), which are proposed to be involved in the transcription elongation, were essential. Strikingly, *M164_1885*, coding for an orthologue of the eukaryotic transcriptional elongation factor Elf1 found in all Crenarchaeota[54], was non-essential. Functional characterization of the *elf1* deletion mutant in *S. islandicus* will help us to understand the roles of *elf1* in archaeal transcription. Four paralogues of putative transcript cleavage factor (TFS1, TFS2, TFS3, and TFS4) were found in the genome of *S. solfataricus* and *S. islandicus*[55], among which *tfs1* (*M164_1859*), *tfs3* (*M164_1858*), and *tfs4* (*M164_0715*) were predicted to be non-essential by Tn-seq analysis. This prediction was confirmed via successful obtainment of individual knockout mutants in standard growth conditions (Supplementary Fig. 3b). The fourth *tfs2* (*M164_1524*), highly conserved in all Crenarchaeota, was classified as “unassigned” by Tn-seq analysis; however, it was later confirmed to be essential by means of knockout analysis, suggesting TFS2 plays more crucial functions in comparison to the other three TFS paralogs.

Unlike the euryarchaeon *H. volcanii* in which only a single SmAP is encoded[56], crenarchaea contain three SmAP paralogues (hereafter named as SmAP1, SmAP2, and SmAP3) annotated as “small nuclear ribonucleoprotein (snRNP) homolog”[57]. Tn-seq analysis revealed *smAP1*

(*M164_1376*) and *smAP2* (*M164_1942*) were essential whereas *smAP3* (*M164_1873*) was non-essential. The essentiality of both *smAP1* and *smAP2* genes could be possibly explained by a previous study, which demonstrated that SmAP1/SmAP2 strongly interacted with each other and co-purified with essential components involved in exosome, RNA modification, turnover, and translation[58]. In eukaryotes, the biogenesis of spliceosomal snRNP proteins required involvement of the SMN protein that interacted with an evolutionarily conserved zinc finger protein ZPR1[59]. Targeted disruption studies of *zpr1* indicated that it was essential for cell viability[60], and played important roles in transcription and cell cycle[61]. *M164_0237*, encoding a homolog of ZPR1, was an essential gene candidate in our Tn-seq assay, but its assignment was inconclusive in *M. maripaludis*[1]. Although categorized as "general functional prediction only" in the arCOG functional database, it is tempting to speculate ZPR1 plays a similar role in Crenarchaeota. The remaining essential genes in this functional category were mostly annotated as transcription regulators with unknown specific functions.

3. Translation

Tn-seq analysis revealed that 113 were essential among 200 genes in arCOG functional category [J]), mostly composed of ribosomal proteins, aminoacyl-tRNA synthetases (aaRSs), and translation initiation/elongation factors. Ribosomal proteins in *S. islandicus* M.16.4 are composed of 37 large- and 28 small subunits, among which 27 large-subunit encoding genes and 20 small-subunit encoding genes were essential. Two small-subunit ribosomal proteins M164_1730 and M164_1557, homologs of the archaeon-eukaryote S25e and S26e, respectively, were non-essential in *S. islandicus* M.16.4. The third, *M164_1159*, encoding the small-subunit ribosomal protein S27e, was non-essential though it is widely distributed in the archaeal domain.

Genomic analysis revealed 21 aaRS-related genes were present in *S. islandicus* M.16.4, among which 18 genes were essential. Two genes, *M164_0290* (hereafter named as *thrS1*) and *M164_1768* (hereafter named as *thrS2*), encode ThrRS in *S. islandicus* M.16.4. The *thrS1* was classified as "unassigned" whereas *thrS2* was non-essential. Further genetic analysis showed that *thrS1* could not be knocked out; however, the *thrS2* disruption mutant could be readily generated (Supplementary Fig. 3b), suggesting that *thrS1* plays a crucial function in protein synthesis. Two genes (*M164_1539* and *M164_1649*; named as *leuS1* and *leuS2* respectively) encoding LeuRS were annotated in *S. islandicus* M.16.4; however, only *leuS1* was required for cell survival, leaving the function of *leuS2* unknown. Notably, the two freestanding homologues of AlaRS editing domain, AlaX1 (M164_1702) and AlaX2 (M164_0462), shown to hydrolyze misacylated $tRNA^{Ala}$ in *S. solfataricus*[62], were non-essential in *S. islandicus* M.16.4. This

finding was further confirmed by genetic analysis (Supplementary Fig. 3b), indicating that they have overlapped functions or play less fundamental roles. *S. islandicus* M.16.4 possesses all aaRSs required for synthesizing each aminoacyl-tRNA except for GlnRS and AsnRS, which are used to directly attach Gln and Asn respectively to their cognate tRNAs. These observations indicate that aminoacyl-tRNA amidotransferase (Adt) is required for the synthesis of Gln-tRNA and Asn-tRNA. Comparative genomic analysis showed that *S. islandicus* M.16.4 contained two types of Adt, which are supposed to correct the misacylated Glu-tRNA^{Gln} and/or Asp-tRNA^{Asn} in the indirect pathway of Gln-tRNA^{Gln} and or/ Asn-tRNA^{Asn}. The first Adt (Asp/Glu-Adt), existing in most bacteria and some archaea and capable of synthesizing both Asn-tRNA and Gln-tRNA[63], is supposed to function as a heterotrimeric enzyme (GatCAB) similarly in *S. islandicus* M.16.4. Though three GatA paralogues (named as GatA-1, 2, and 3) are present, only *gatA*-1 (*M164_1253*) is essential, suggesting that *gatA*-2 (*M164_0374*) or *gatA*-3 (*M164_1369*) is functionally redundant. The other two subunits (GatB and GatC) of GatCAB encoded by *M164_1911* and *M164_1252* respectively were essential as revealed by Tn-seq analysis. Additionally, *S. islandiucs* M1.6.4 possesses a heterodimeric amidotransferase (GatDE) for Gln-tRNA^{Gln} formation, which has been biochemically characterized in *Methanothermobacter thermautotrophicus* and predicted to be exclusively Archaea-specific[64,65]. The GatD and GatE subunits, encoded by two adjacent genes *M164_1273* (annotated as *ansB* and assigned into arCOG functional category [E]) and *M164_1274*, respectively, were essential in *S. islandicus* M.16.4. The existence and essentiality of both GatCAB and GatDE in *S. islandicus* suggest these two complexes play distinct functions in protein synthesis.

All 13 genes involved in the cycle of translation were essential in *S. islandicus* M.16.4. These include 9 translation initiation factors: aIF-1A (M164_0191), aIF-2 (M164_1916), a/eIF2α (M164_1158), a/eIF2β (M164_0194), a/eIF2γ (M164_1739), aSUI1 (M164_1707), aIF5A (M164_1237), aIF-6 (M164_1802), and RLI1 (M164_1861), 3 translation elongation factors: EF-1α (M164_1926), EF-1β (M164_1968), and EF-2/EF-G (M164_1407), and one translation termination factor aRF1 (M164_0157). Notably, Sulfolobales do not contain the selenocysteine-specific translation elongation factor (SelB) which extensively exists in *Methanococcales*[66] and was shown to be essential previously in *M. maripaludis*[1]. Instead, a SelB-like protein (SelBL) is present in *S. islandicus* M.16.4 (M164_1681) and found to be widely distributed in diverse archaea[66]. The function of SelBL remains elusive; however, both Tn-seq and genetic analyses (Supplementary Fig. 3b) showed that *selBL* was non-essential, suggesting it plays less fundamental roles in *Sulfolobus* translation.

4. Cell cycle, cell division, and chromosome segregations

In the arCOG functional category [D], six genes were essential inferred by Tn-seq data, including the *cdvA* (*M164_1293*), *cdvB* (*M164_1294*; also named as *escrt-III*), and *cdvC* (*M164_1295*; also named as *vps4*), which have been proved to be the crucial components of ESCRT (Endosomal Sorting Complex Required for Transport)-III-based cell division apparatus in *Sulfolobus*[67,68]. Furthermore, repeated attempts to knock out individual *cdvA*, *cdvB*, and *cdvC* genes in *S. islandicus* M.16.4 failed to generate any transformants in standard growth conditions, further confirming the essentiality of the ESCRT-III system for *Sulfolobus* cell survival. Additionally, like *S. acidocaldarius* and *S. solfataricus* P2, *S. islandicus* M.16.4 contains three *cdvB* paralogs: *cdvB1* (*M164_1700*), *cdvB2* (*M164_1319*), and *cdvB3* (*M164_1510*). Interestingly, the essentiality and function of these three CdvB paralogs seemed to be divergent in *S. acidocaldarius* and *S. islandicus*. Genetic analyses of *cdvB* paralogous genes in *S. acidocaldarius* indicated that none was essential for cell viability though a significant growth defect and impaired cell division were observed in the *cdvB3* mutant[69]. By contrast, a recent genetic study in *S. islandicus* REY15A showed growth between the *cdvB3* deletion mutant and parental strain was indistinguishable, and CdvB3 actually played a role in virus budding rather than cell division[70]. It should be noted that the *cdvB3* in our Tn-seq analysis is possibly an example of false positive essential gene calling, because we can readily obtain the *cdvB3* disruption mutant in standard laboratory conditions (Supplementary Fig. 3b) with a recently developed microhomology-mediated gene inactivation system[71]. Our Tn-seq and genetic knockout analyses confirmed that *cdvB2* was essential in *S. islandicus* M.16.4, in agreement with a previous study demonstrating *cdvB2* was essential and played crucial roles in the late stages of cell division in *S. islandicus* REY15A[70]. In contrast, the *cdvB1* gene, which has been shown to be essential and involved in the early stage of cell division in *S. islandicus* REY15A[70], was very unlikely essential in our strain as validated by both Tn-seq (log_2FC= 0.48; EI=18) and genetic knockout analyses (Supplementary Fig. 3b).

Sulfolobus species utilize a hybrid DNA-partition machine, consisting of two interacting components SegA and SegB, to drive chromosome segregation during M phase of the cell cycle [72]. Unexpectedly, Tn-seq analysis revealed *segA* (*M164_2088*; arCOG functional category [D]) and *segB* (*M164_2087*; arCOG functional category [S]) were non-essential in *S. islandicus* M.16.4, in agreement with genetic knockout experiments (Supplementary Fig. 3b). Further phenotypic characterization of the mutant strains with in-frame deletion in *segA*, *segB*, and *segAB* will help us to dissect the mechanism of chromosome segregation system in *Sulfolobus* species. The remaining essential gene in arCOG functional category [D] *M164_1692* encodes an ATPase-like protein conserved in all three domains.

5. Gene essentiality in central carbon metabolism (CCM)

We also examined gene essentiality in pathways of CCM, including glycolysis, gluconeogenesis, and the oxidative TCA cycle, which have been well reconstructed in a related species *S. solfataricus* P2[73].

Among the genes in glycolysis and gluconeogenesis predicted by the reconstructed central metabolic pathways in *S. solfataricus*[73] and KEGG pathway database, five genes were possibly essential as revealed by Tn-seq data. Among those, three genes, encoding enzymes fructose-bisphosphatase (M164_1862), glucose-6-phosphate isomerase (M164_0092), and phosphoglucomutase (M164_1935), respectively, were involved in the last three steps of gluconeogenesis. The fourth, *M164_2166*, encodes the 2-keto-3-deoxy-(6-phospho) gluconate aldolase (KDG aldolase). The remaining candidate essential gene involved in glycolysis/gluconeogenesis was *M164_2749*, encoding the alpha subunit of 2-oxoacid: ferredoxin oxidoreductase (OFOR) that was presumably responsible for the formation of acetyl-CoA from pyruvate.

Next, we surveyed the essentiality of genes that involved in the reversed ribulose-monophosphate pathway (RuMP), a pathway that substitutes the classic pentose phosphate pathway (PPP) in most of archaea including *S. islandicus* M.16.4. Like other *Sulfolobus* species[74], *S. islandicus* M.16.4 contains all enzymes involved in the RuMP pathway, including 6-phospho-3-hexuloisomerase (M164_1993), 3-hexulose-6-phosphate synthase (M164_1939), ribose-5-phosphate isomerase (M164_1228), ribose-phosphate pyrophosphokinase (M164_1165), and transketolase fused by two subunits (M164_1848/M164_1849). Our Tn-seq data showed that all six genes were essential, suggesting that the RuMp pathway was indispensable for cell survival in *S. islandicus* under standard laboratory conditions.

Comparative genomic analysis showed that *S. islandicus* M.16.4 harbors a complete TCA cycle. The candidate enzymes for all steps of TCA cycle were present except for the 2-oxoglutarate dehydrogenase complex (OGDC). Considering the absence of OGDC and the broad substrate specificity of OFOR towards 2-oxoacids[73,75], formation of succinyl-CoA from 2-oxoglutarate in the TCA cycle was proposed to be operated by the OFOR in *S. islandicus* M.16.4 as well. There are 13 genes encoding for core enzymes of the TCA cycle in *S. islandicus* M.16.4, 12 of which were shown to be essential, whereas the remaining one, *M164_2478*, encoding the beta subunit of SisOFOR, was classified as "unassigned". Notably, three other SisOFOR (*M164_2479*/*M164_2478*) paralogous gene pairs i.e. *M164_0365*/*M164_0364*, *M164_0396*/*M164_0395*, and *M164_2553*/*M164_2552* are present in *S. islandicus* M.16.4, all of which were non-essential. These observations and our experimental data demonstrated that

physiological roles of SisOFOR (*M164_2479*/*M164_2478*) were irreplaceable in the TCA cycle. Lastly, our Tn-seq data revealed that *M164_0683* and *M164_0684*, encoding isocitrate lyase and malate synthase, respectively, were non-essential, indicating the glyoxylate cycle was not required in our laboratory conditions.

Supplementary Tables

Supplementary Table 1: Summary of number of reads and insertions in three independent transposon mutant libraries

Library	No. of colonies	Colony collection way	No. of reads mapped to the genome	No. of unique insertions in total	No. of unique insertions(>1 reads)	No. of unique insertions(>2 reads)	No. of unique insertions(>3 reads)
CYZ-TL1	25, 318	Pick	2.9×10^7	83, 906	30, 210	23, 948	22, 539
CYZ-TL2	36, 840	Wash	1.7×10^7	47, 967	34, 664	33, 881	33, 582
CYZ-TL2	43, 810	Wash	2.5×10^6	35, 217	33, 712	33, 132	32, 640
Total	105, 968	-	-	167, 090	98, 586	90, 961	88, 761

Supplementary Table 2: Summary of self-nucleotide BLAST results that reveal matching sections elsewhere in the genome

Locus tag	Start	End	Match start	Match end	Match length (bp)	Match ID%	Genes hit
M164_0862	808672	809127	2010585	2010442	144	97.22	None
M164_1012	952035	951631	559226	558828	406	83.5	M164_0624
M164_1867	1712920	1711262	1264800	1264914	115	86.09	M164_1334

Supplementary Table 3: Evaluation of selected essential/non-essential gene candidates inferred by Tn-seq, and then confirmed with genetic knockout analysis in *S. islandicus*

Cellular Process	Gene symbols/locus tags[#]	Essentiality by Tn-seq	Essentiality by KO assay[##]	Source/Reference
Replication	*orc1-1, orc1-2, orc1-3, whip*	-	-	[76]
	mcm, gins23, gins15, priS, rpa, dpoB1, rfcL, rfcS, fen1, cdc45, nrdJ, dnaG, pbp2	+	+	This study and[34]
	pcna1, pcna2, pcna3	+	+	[13]
	lig, priL, priX, rnhII	Unassigned	+	This study and[17]
	dpoB2, dpoB3, dpo4, pbp1, nrdB	-	-	This study
Recombination /Repair	*nurA, rad50, mre11, herA, radA*	+	+	[34]
	radB, radC1, radC2	-	-	[34,77]
	hje, hjc	-	-	[78]
	hjm (*hel308a*), *xer*	-	-	This study
	xpb1, xpb2, xpd, xpf, bax1, phrB	-	-	[34]
	endoMS, ogt, ogg2, udg5	-	-	This study
	exoIII, endoIII, endoIV, endoV	-	-	This study and[79]
	udg4	Unassigned	-	This study
Chromatin	*topR1*	-	-	This study
	topR2	+	-	This study
	topIII (*topIA*)	-	-	[80]
	top6A, top6B	+	+	This study
	cren7, alba1	+	+	This study
	sul7d1, sul7d2, alba2, sir2, pat	-	-	This study
Cell division /genome segregation	*cdvA, cdvB, cdvC, cdvB2*	+	+	This study
	cdvB3	+	-	This study
	cdvB1	-	-	This study

	segA, *segB*	-	-	This study
Transcription	*tbp*, *tfb1*, *tfb2*, *tfe-α*, *tfe-β*, *nusA*, *nusA-like*, *nusG*, *spt4*	+	+	This study
	tfs2	Unassigned	+	This study
	rpo8	Unassigned	-	This study
	tfb3, *tfs1*, *tfs3*, *tfs4*, *tip49*, *rpo13*	-	-	This study
Translation	*alaX1*, *alaX2*, *leuS2*, *thrS2*, *selBL*	-	-	This study
	thrS1	Unassigned	+	This study
Other functional categories	*lacS*, *pyrE*, *pyrF*, *amyA*, *upsE*, *upsF*, *cas1*, *cas3'*, *cas3"*, *cas6*, *csa5*, *cas7*, *cmr2a* (*cas10*), *cas4*, *cas2*, *csa1*, *cbp1*, *csa3a**, *csa3b*,	-	-	[81-89]
	cas5	+	+	This study
	pinA	+	+	[90]
	aKMT	-	-	[79]
	M164_0809, *M164_2103*, *M164_2020*	-	-	This study
	M164_1243	+	+	[91]
	M164_1756, *M164_0737*	-	-	[91]
	M164_1060	+	+	This study
	apt	+	-[$]	[83]

+: Essential; -: Non-essential.

[#]: Locus tags and annotations of genes were shown in Supplementary Data 10.

[##]KO assay: Gene knockout experiment was performed at least 4 times for every possibly essential/non-essential gene inferred by Tn-seq. The gene essentiality in the KO assay was determined based on the facts that no transformants or only false positive transformants were obtained in nutrition-rich plates with 10-20 days' incubation at 76-78 °C.

*: The *csa3a* gene that encodes for a transcriptional regulator of *cas* genes[87] in *S. islandicus* M.16.4 is split by an approximate 14 kb of integrated provirus.

[$]: The strain with an inactivation of *apt* gene, encoding the adenine phosphoribosyltransferase, exhibited extremely slow/poor growth on solid plates lacking AMP or GMP[83].

Supplementary Table 4: Comparison of observed phyletic distribution to 100 scaled randomizations in terms of number of genes

Category	# genes observed	Mean # genes simulated	stdev genes simulated	p_{above}	p_{below}	$p_{2\text{-}tail}$
Universal	141	163.83	3.52	0	0	0
EA	80	8.69	2.82	0	0	0
Archaea	55	9.81	2.66	0	0	0
TACK	18	10.39	3.21	0	0	0
Sulfolobales	73	147.09	3.89	0	0	0
Other	74	61.96	5.59	0	0.02	0.02

Notes: P-values are an estimate based on simulated distribution (See "Methods"). Counts in the "Simulated" columns are the arithmetic mean of 100 random counts. Categories are defined

in Table 1.

Supplementary Table 5: Summary of essential genes compared to previously assembled ancient gene sets (a summary of Supplementary Data 8)

Total conserved COGs in *S. islandicus*	Excluded conserved*	Essential conserved†	% Conserved essential‡	References
99	6	67	72%	[92]Puigbò, et al. 2009 NUTs (COGs)
78	3	64	85%	[93]Harris, et al. 2003 LCA (COGs)
236	25	123	58%	[94]Gil, et al. 2003 (COGs)
165	57	56	52%	[95]Weiss, et al. 2016 LUCA (COGs)
931	153	314	40%	[96]Wolf, et al. 2012 LACA (arCOGs)
26	0	25	96%	[97]Guy and Ettema 2011 Universal Genes (COGs)
48	1	36	77%	[98]Raymann, et al. 2015 Archaea/Bacteria (COGs)
73	2	57	80%	[98]Raymann, et al. 2015 Archaea/Eukaryota (COGs)[97]
24	6	10	56%	[99,100]ESPs and membrane remodeling proteins in *Sulfolobus* (arCOGs)
34	0	29	85%	[101]Yutin, et al. 2012 universal ribosomal proteins (arCOGs)
386	70	157	50%	[102]Mirkin, et al. 2003 LUCA (COGs)
111	10	73	72%	[103]Makarova, et al. 2015 (arCOGs)

***** Excluded because of multiple matching gene for COGs.

† At least one *S. islandicus* gene in COG is essential.

‡ Equal to (Essential) / (Total – Excluded).

Supplementary Table 6: Poorly characterized essential genes shared within Archaea, Eukaryotes, and Sulfolobales

Locus tags	Phyletic category	arCOG	Predicted characteristics
M164_1243	Archaea	arCOG00557	Lhr-like helicase with C-terminal Zn finger domain
M164_1908	Archaea	arCOG00933	Radical SAM superfamily enzyme
M164_1554	Archaea	arCOG04116	ATPase (PilT family)
M164_1444	Archaea	arCOG04055	SHS2 domain protein implicated in nucleic acid metabolism
M164_1735	Archaea	arCOG04076	Uncharacterized protein, DUF359 family

M164_1930	Archaea	arCOG01831	Predicted nucleotidyltransferase
M164_1582	Archaea	arCOG01285	OB-fold domain and Zn-ribbon containing protein, possible acyl-CoA-binding protein
M164_1410	Archaea	arCOG04458	Uncharacterized protein of DIM6/NTAB family
M164_1948	Archaea	arCOG04290	PIN-domain and Zn ribbon
M164_1373	Archaea	arCOG00543	Predicted metal-dependent RNase, consists of a metallo-beta-lactamase domain and an RNA-binding KH domain
M164_2044	Archaea	arCOG00932	Uncharacterized protein related to pyruvate formate-lyase activating enzyme
M164_1168	Archaea	arCOG01043	Predicted RNA binding protein with dsRBD fold
M164_1936	Archaea	arCOG04124	Uncharacterized protein, Trm112 family
M164_1350	Archaea	arCOG04308	Uncharacterized protein
M164_0237	Eukaryotes/Archaea	arCOG04265	C4-type Zn-finger protein
M164_0664	Sulfolobales	arCOG01314	Uncharacterized membrane anchored protein with extracellular flavodoxin-like domain, a component of a putative secretion system
M164_2107	Sulfolobales	arCOG05396	Uncharacterized membrane protein
M164_1303	Sulfolobales	arCOG08333	Uncharacterized protein
M164_1025	Sulfolobales	arCOG08451	Uncharacterized protein
M164_1520	Sulfolobales	arCOG05995	Uncharacterized protein
M164_0149	Sulfolobales	arCOG07185	Uncharacterized protein
M164_1645	Sulfolobales	arCOG05923	Uncharacterized protein
M164_1726	Sulfolobales	arCOG05939	Uncharacterized protein
M164_1958	Sulfolobales	arCOG08308	Uncharacterized protein
M164_0627	Sulfolobales	arCOG03239	ATPase, predicted component of phage defense system
M164_0066	Sulfolobales	arCOG01098	Uncharacterized protein
M164_1338	Sulfolobales	arCOG05983	Uncharacterized protein
M164_0682	Sulfolobales	arCOG08424	Uncharacterized protein
M164_1275	Sulfolobales	arCOG05980	Uncharacterized protein

M164_0178	Sulfolobales	arCOG00442	von Willebrand factor type A (vWA) domain containing protein
M164_2177	Sulfolobales	arCOG05926	Uncharacterized protein
M164_0169	Sulfolobales	arCOG05958	Uncharacterized protein
M164_2151	Sulfolobales	arCOG03699	Uncharacterized membrane protein
M164_1620	Sulfolobales	arCOG09897	Uncharacterized protein
M164_0677	Sulfolobales	arCOG13101	Uncharacterized protein
M164_1289	Sulfolobales	arCOG04323	Zn-finger protein
M164_1789	Sulfolobales	arCOG06088	Zn finger protein
M164_1865	Sulfolobales	arCOG07188	Uncharacterized protein
M164_1724	Sulfolobales	arCOG05941	Uncharacterized protein
M164_1345	Sulfolobales	arCOG07185	Uncharacterized protein
M164_0089	Sulfolobales	arCOG05899	Cell surface protein
M164_1302	Sulfolobales	arCOG04103	Zn finger protein
M164_2100	Sulfolobales	arCOG01830	Predicted nucleotidyltransferase
M164_2636	Sulfolobales	arCOG06032	Uncharacterized membrane protein, DUF1404 family
M164_0727	Sulfolobales	arCOG10132	Uncharacterized protein
M164_1483	Sulfolobales	arCOG05997	Uncharacterized protein
M164_0224	Sulfolobales	arCOG05950	Uncharacterized protein
M164_0254	Sulfolobales	arCOG05929	Uncharacterized protein
M164_1337	Sulfolobales	arCOG06043	Uncharacterized protein
M164_0165	Sulfolobales	arCOG07197	Uncharacterized membrane protein
M164_0246	Sulfolobales	arCOG08319	Uncharacterized protein
M164_2845	Sulfolobales	arCOG07934	Uncharacterized protein
M164_1032	Sulfolobales	arCOG07229	Uncharacterized protein
M164_0037	Sulfolobales	arCOG05885	Uncharacterized protein
M164_2723	Sulfolobales	arCOG05922	Uncharacterized protein
M164_1572	Sulfolobales	arCOG04251	Uncharacterized protein
M164_2767	Sulfolobales	arCOG03031	Chlorite dismutase
M164_1332	Sulfolobales	arCOG04160	Uncharacterized protein
M164_0185	Sulfolobales	arCOG05956	Metal-binding protein with CxxC..HxxxxH signature
M164_0756	Sulfolobales	arCOG07185	Uncharacterized protein
M164_1251	Sulfolobales	arCOG07217	Uncharacterized protein

Supplementary Table 7: Strains and plasmids used in this study

**Strains and plasmids**	**Genotypes/Descriptions**	**Reference /Source**
**Strains**		
S. islandicus M.16.4	Wild type	[104]
S. solfataricus P2	Wild type	DSMZ
S. islandicus RJW004	△*pyrEF*△*lacS*△*argD*; Derived from *S. islandicus* M.16.4	[81]
S. islandicus RJW008	△*argD*; derived from *S. islandicus* M.16.4	[105]
S. islandicus RJW011	RJW004△*slaA* (△*slaA*); *slaA* was deleted from RJW004 via in-frame deletion	This study
S. islandicus RJW012	RJW004△*slaB* (△*slaB*); *slaB* was deleted from RJW004 via in-frame deletion	This study
S. islandicus RJW013	RJW004△*slaAB* (△*slaAB*); *slaA* and *slaB* were deleted from RJW004 via in-frame deletion	This study
S. islandicus △*slaAB* △*M 164_1049*	△*pyrEF*△*lacS*△*argD*△*slaAB*△*M 164_1049*:: *StoargD*; derived from RJW013	This study
S. islandicus △*dpoB2*	△*argD*△*dpoB2::StoargD*; derived from RJW008	This study
S. islandicus △*dpoB3*	△*argD*△*dpoB3::StoargD*; derived from RJW008	This study
S. islandicus △*dpo4*	△*argD*△*dpo4::StoargD*; derived from RJW008	This study
S. islandicus △*pbp1*	△*argD*△*pbp1::StoargD*; derived from RJW008	This study
S. islandicus △*topR1*	△*argD*△*topR1::StoargD*; derived from RJW008	This study
S. islandicus △*topR2*	△*argD*△*topR2::StoargD*; derived from RJW008	This study
S. islandicus △*udg4*	△*argD*△*udg4::StoargD*; derived from RJW008	This study
S. islandicus △*udg5*	△*argD*△*udg5::StoargD*; derived from RJW008	This study
S. islandicus △*endoIII*	△*argD*△*endoIII::StoargD*; derived from RJW008	This study
S. islandicus △*endoV*	△*argD*△*endoV::StoargD*; derived from RJW008	This study
S. islandicus △*ogt*	△*argD*△*ogt::StoargD*; derived from RJW008	This study
S. islandicus △*ogg2*	△*argD*△*ogg2::StoargD*; derived from RJW008	This study
S. islandicus △*nrdB*	△*argD*△*nrdB::StoargD*; derived from RJW008	This study
S. islandicus △*sir2*	△*argD*△*sir2::StoargD*; derived from RJW008	This study
S. islandicus △*pat*	△*argD*△*pat::StoargD*; derived from RJW008	This study
S. islandicus △*alba2*	△*argD*△*alba2::StoargD*; derived from RJW008	This study
S. islandicus △*sul7d1*	△*argD*△*sul7d1::StoargD*; derived from RJW008	This study
S. islandicus △*sul7d2*	△*argD*△*sul7d2::StoargD*; derived from RJW008	This study
S. islandicus △*segA*	△*argD*△*segA::StoargD*; derived from RJW008	This study
S. islandicus △*segB*	△*argD*△*segB::StoargD*; derived from RJW008	This study
S. islandicus △*xer*	△*argD*△*xer::StoargD*; derived from RJW008	This study
S. islandicus △*hjm*	△*argD*△*hjm::StoargD*; derived from RJW008	This study
S. islandicus △*endoMS*	△*argD*△*endoMS::StoargD*; derived from RJW008	This study

S. islandicus △*cdvB1*	△*argD*△*cdvB1::StoargD*; derived from RJW008	This study
S. islandicus △*cdvB3*	△*argD*△*cdvB3::StoargD*; derived from RJW008	This study
S. islandicus △*tfb3*	△*argD*△*tfb3::StoargD*; derived from RJW008	This study
S. islandicus △*M164_0809*	△*argD*△*M164_0809::StoargD*; derived from RJW008	This study
S. islandicus △*M164_2020*	△*argD*△ *M164_2020::StoargD*; derived from RJW008	This study
S. islandicus △*M164_2103*	△*argD*△ *M 164_2103::StoargD*; derived from RJW008	This study
S. islandicus △*tip49*	△*argD*△*tip49::StoargD*; derived from RJW008	This study
S. islandicus △*tfs1*	△*argD*△*tfs1::StoargD*; derived from RJW008	This study
S. islandicus △*tfs3*	△*argD*△*tfs3::StoargD*; derived from RJW008	This study
S. islandicus △*tfs4*	△*argD*△*tfs4::StoargD*; derived from RJW008	This study
S. islandicus △*rpo8*	△*argD*△*rpo8::StoargD*; derived from RJW008	This study
S. islandicus △*rpo13*	△*argD*△*rpo13::StoargD*; derived from RJW008	This study
S. islandicus △*alaX1*	△*argD*△*alaX1::StoargD*; derived from RJW008	This study
S. islandicus △*alaX2*	△*argD*△*alaX2::StoargD*; derived from RJW008	This study
S. islandicus △*selBL*	△*argD*△*selBL::StoargD*; derived from RJW008	This study
S. islandicus △*leuS2*	△*argD*△*leuS2::StoargD*; derived from RJW008	This study
S. islandicus △*thrS2*	△*argD*△*thrS2::StoargD*; derived from RJW008	This study
**Plasmids**		
pMOD^{TM}-2 <MCS>	Transposon construction vector	Epicentre, USA
pT-SsoargD	pMOD^{TM}-2 <MCS> carrying an *argD* expression cassette derived from *S. solfataricus* P2	This study
pSeSd	*Sulfolobus-E. coli* shuttle vector	[106]
pSeSd-SsoargD	pSeSd carrying an *argD* expression cassette derived from *S. solfataricus* P2	This study
pSeSd-StoargD	pSeSd carrying an *argD* expression cassette derived from *S. tokodaii*	[105]
pRJW8	pUC19 carrying a triple marker gene cassette *pyrEF-lacS-argD* derived from *S. solfataricus* P2; cloning vector	[81]
pMID-slaA	pRJW8 carrying Up-arm and Dn-arm of *slaA* and a partial region of *slaA* (Tg-arm); *slaA* knockout plasmid	This study
pMID-slaB	pRJW8 carrying Up-arm and Dn-arm of *slaA* and a partial region of *slaB* (Tg-arm); *slaB* knockout plasmid	This study
pMID-slaAB	pRJW8 carrying Up-arm of *slaA*, Dn-arm of *slaB*, and a partial region of *slaB* (Tg-arm); *slaAB* knockout plasmid	This study

Supplementary Table 8: Expected sizes of amplicons generated from the genetic host (Wt) and mutant strains using two different primer sets

				Flanking primers		Internal primers	
Gene name	Gene length (bp)	Deletion region (bp)	Replace region (bp)	Amplicon size (bp)		Amplicon size (bp)	
				Wt	Mutant	Wt	Mutant
dpoB2	1668	1276	740	1836	1300	990	0
dpoB3	2292	1858	740	2395	1277	868	0
dpo4	1059	897	740	1168	1011	547	0
pbp1	294	189	740	554	1105	155	0
topR1	3720	3520	740	3996	1216	777	0
topR2	3501	3250	740	3788	1278	638	0
slaA#	3690	3636	6	5870	2240	740	0
slaB#	1194	1173	6	3193	2026	540	0
slaAB#	N.A$	4865	6	6973	2114	2014	0
M164_1049	1803	1761	740	2109	1046	1159	0
udg4	651	466	740	897	1171	312	0
udg5	669	613	740	847	974	327	0
endoIII	702	532	740	778	986	375	0
endoV	597	481	740	743	1002	336	0
ogt	456	413	740	643	970	397	0
ogg2	624	467	740	697	970	277	0
nrdB	927	873	740	1116	983	560	0
sir2	744	587	740	830	983	352	0
pat	483	422	740	647	965	251	0
alba2	270	228	740	477	989	181	0
sul7d1	195	195	740	370	915	N.D*	N.D*
sul7d2	195	194	740	457	1003	N.D*	N.D*
M164_0809	2001	1810	740	2319	1249	1012	0
M164_2020	2628	2449	740	2827	1118	965	0
M164_2103	2226	1887	740	2262	1115	769	0
segA	663	594	740	865	1011	383	0
segB	330	253	740	554	1041	201	0
cdvB1	762	490	740	755	1005	380	0
tfb3	513	431	740	895	1204	250	0
hjm	2148	2009	740	2354	1085	780	0
xer	876	842	740	1017	915	598	0
endoMS	732	635	740	934	1039	502	0
cdvB3	507	436	740	843	1147	301	0
tfs1	336	276	740	694	1158	254	0
tfs3	273	215	740	546	1071	185	0
tfs4	228	180	740	610	1170	108	0
tip49	1359	1227	740	1771	1284	569	0
rpo13	315	180	740	615	1175	164	0
rpo8	399	310	740	727	1157	266	0
selBL	906	846	740	1026	920	553	0
leuS2	2805	2507	740	2902	1135	1166	0
thrS2	1161	1018	740	1554	1276	680	0
alaX1	711	621	740	846	965	350	0
alaX2	453	370	740	811	1181	327	0

Deletions of *slaA*, *slaB*, and *slaAB* were achieved via a markerless in-frame deletion approach.

N.A$: Not applied.

N.D*: Not determined.

Supplementary Figures and Legends

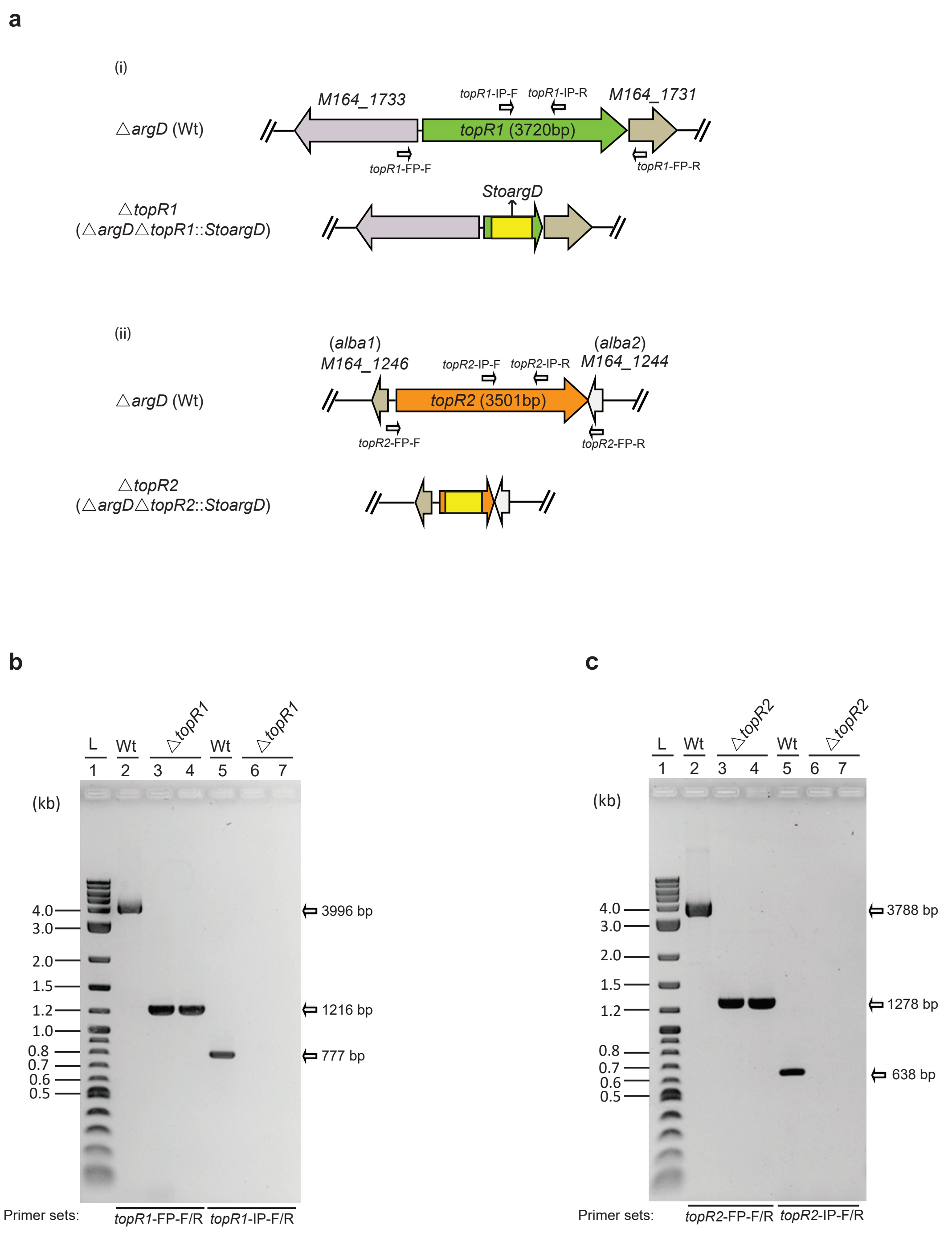

Supplementary Figure 1: Confirmation of genotypes of reverse gyrase disruption mutants. **a**, Genomic context of *topR1* and *topR2* in the genetic host (Wt) and mutant strains. **b**, PCR verification of △*topR1* mutant strain. **c**, PCR verification of △*topR2* mutant strain. L indicates 2-Log DNA Ladder (NEB, USA) and sizes of DNA bands are labelled.

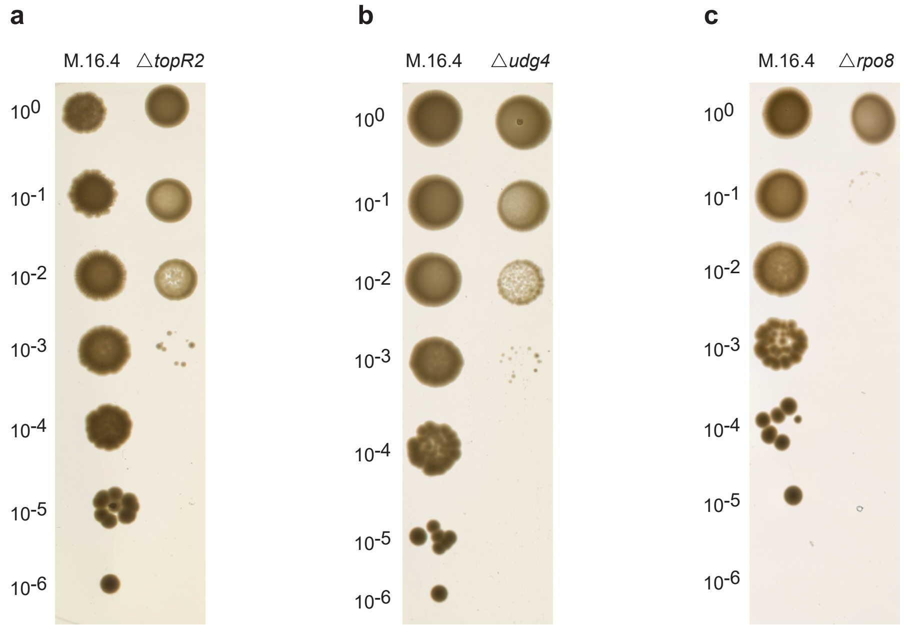

Supplementary Figure 2: Disruption of *topR2*, *udg4*, and *rpo8* reduced cell viability. The *S. islandicus* M.16.4 (agmatine prototrophy), △*topR2* (△*argD*△*topR2*::*StoargD*), △*udg4* (△*argD*△*udg4*::*StoargD*), and △*rpo8* (△*argD*△*rpo8*::*StoargD*) strains were grown in DY liquid medium at 76 °C. Cell cultures at the mid-log phase were normalized to OD_{600}=0.5, and then serially diluted ten fold with 1× DY. Ten microliter of diluted cells were spotted on DY plates and then incubated at 76°C for 12 days. The resulting spots are imaged using an EPSON scanner.

a

b

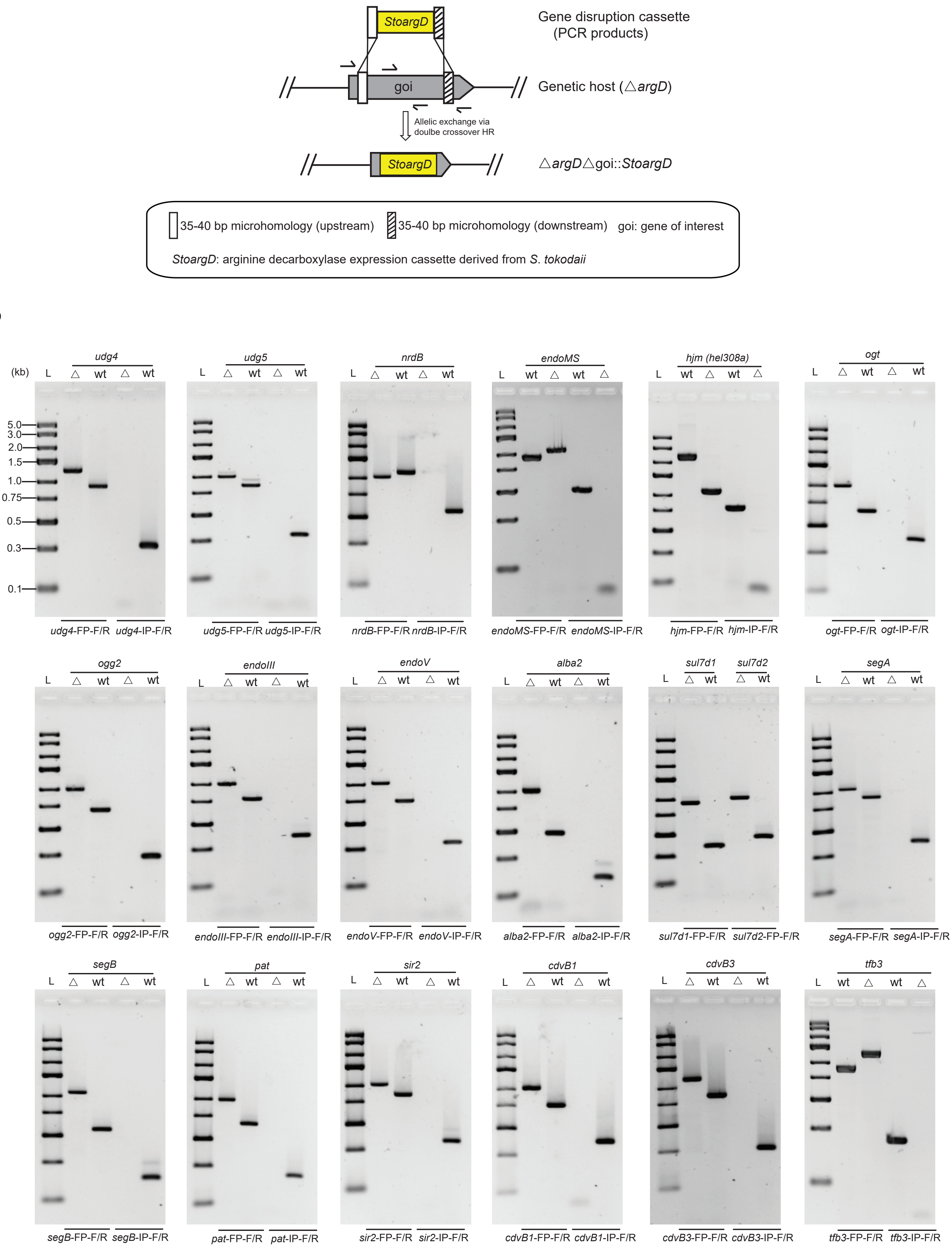

StoargD
Gene disruption cassette
(PCR products)
goi
Genetic host (△argD)
Allelic exchange via
doulbe crossover HR
StoargD
△argD△goi::StoargD
35-40 bp microhomology (upstream)
35-40 bp microhomology (downstream)
goi: gene of interest
StoargD: arginine decarboxylase expression cassette derived from S. tokodaii
(kb)
5.0
3.0
2.0
1.5
1.0
0.75
0.5
0.3
0.1
L
△
wt
udg4
udg4-FP-F/R
udg4-IP-F/R
udg5
udg5-FP-F/R
udg5-IP-F/R
nrdB
nrdB-FP-F/R
nrdB-IP-F/R
endoMS
endoMS-FP-F/R
endoMS-IP-F/R
hjm (hel308a)
hjm-FP-F/R
hjm-IP-F/R
ogt
ogt-FP-F/R
ogt-IP-F/R
ogg2
ogg2-FP-F/R
ogg2-IP-F/R
endoIII
endoIII-FP-F/R
endoIII-IP-F/R
endoV
endoV-FP-F/R
endoV-IP-F/R
alba2
alba2-FP-F/R
alba2-IP-F/R
sul7d1
sul7d2
sul7d1-FP-F/R
sul7d2-FP-F/R
segA
segA-FP-F/R
segA-IP-F/R
segB
segB-FP-F/R
segB-IP-F/R
pat
pat-FP-F/R
pat-IP-F/R
sir2
sir2-FP-F/R
sir2-IP-F/R
cdvB1
cdvB1-FP-F/R
cdvB1-IP-F/R
cdvB3
cdvB3-FP-F/R
cdvB3-IP-F/R
tfb3
tfb3-FP-F/R
tfb3-IP-F/R

b (Continued)

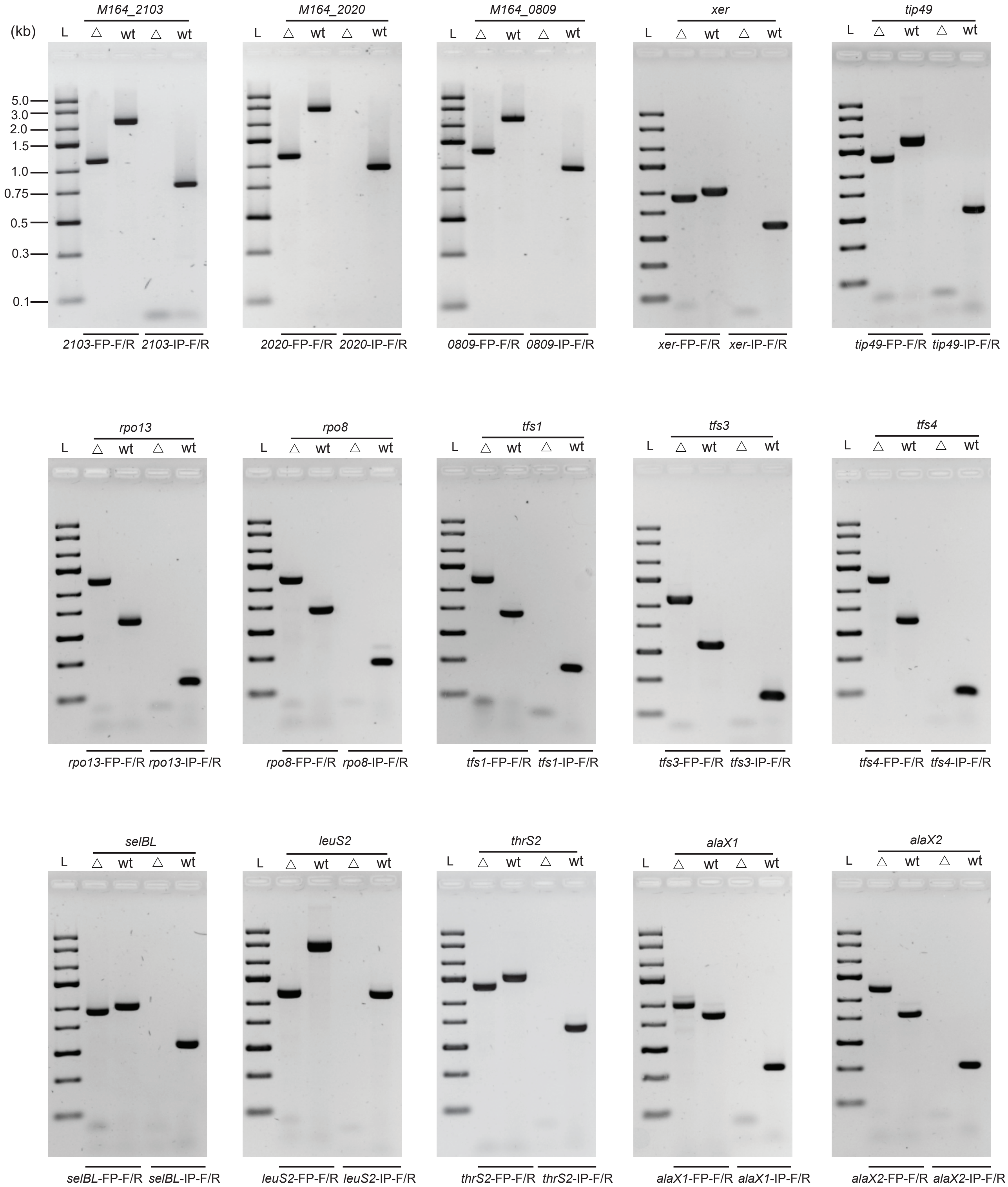

M164_2103
M164_2020
M164_0809
xer
tip49
(kb)
L
△ wt △ wt
5.0
3.0
2.0
1.5
1.0
0.75
0.5
0.3
0.1
2103-FP-F/R 2103-IP-F/R
2020-FP-F/R 2020-IP-F/R
0809-FP-F/R 0809-IP-F/R
xer-FP-F/R xer-IP-F/R
tip49-FP-F/R tip49-IP-F/R
rpo13
rpo8
tfs1
tfs3
tfs4
rpo13-FP-F/R rpo13-IP-F/R
rpo8-FP-F/R rpo8-IP-F/R
tfs1-FP-F/R tfs1-IP-F/R
tfs3-FP-F/R tfs3-IP-F/R
tfs4-FP-F/R tfs4-IP-F/R
selBL
leuS2
thrS2
alaX1
alaX2
selBL-FP-F/R selBL-IP-F/R
leuS2-FP-F/R leuS2-IP-F/R
thrS2-FP-F/R thrS2-IP-F/R
alaX1-FP-F/R alaX1-IP-F/R
alaX2-FP-F/R alaX2-IP-F/R

b (Continued)

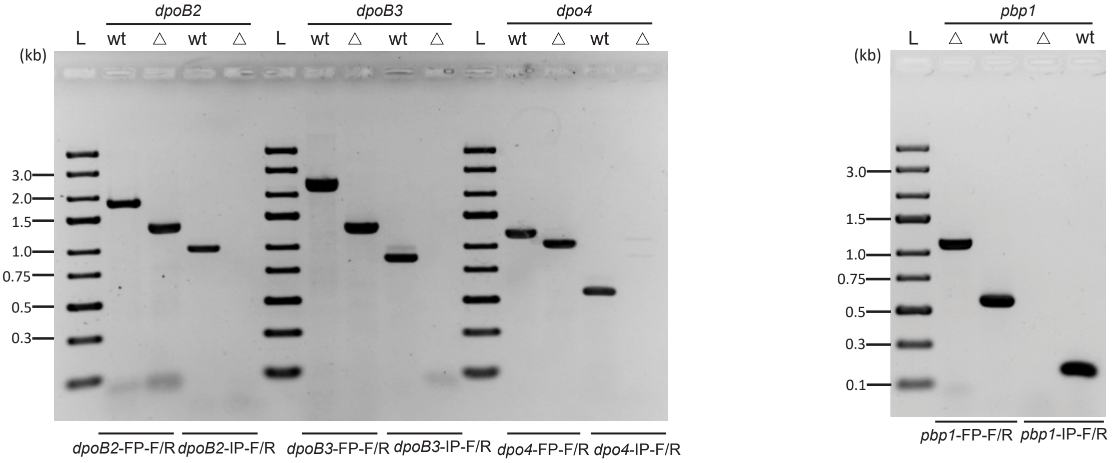

Supplementary Figure 3: Verification of selected non-essential genes via a microhomology-mediated gene inactivation approach (MMGI) in *S. islandicus*[105]. a, Schematic illustration of the MMGI. The gene disruption cassette, consisting of the *StoargD* marker flanked by 35-40 bp of microhomology that corresponded to flanking sequences of the targeted region, was transformed into an ArgD$^-$ strain, yielding ArgD$^+$ colonies via a double-crossover HR event. **b,** Confirmation of *S. islandicus* gene disruptions (Related to Supplementary Table 3 and Supplementary Data 10) by PCR analyses of target gene locus. A flanking primer set (-FP-F/R) annealing to upstream and downstream sequences of the HR regions of goi, and an internal primer set (-IP-F/R) specifically binding to the coding region of goi, were used to confirm the gene disruptions. L indicates the GeneRuler Express DNA Ladder (Thermo Fisher, USA) and the marker sizes are labelled. The wt and △ denote the parental strain and mutant strain respectively. The expected sizes of amplicons are shown in Supplementary Table 8.

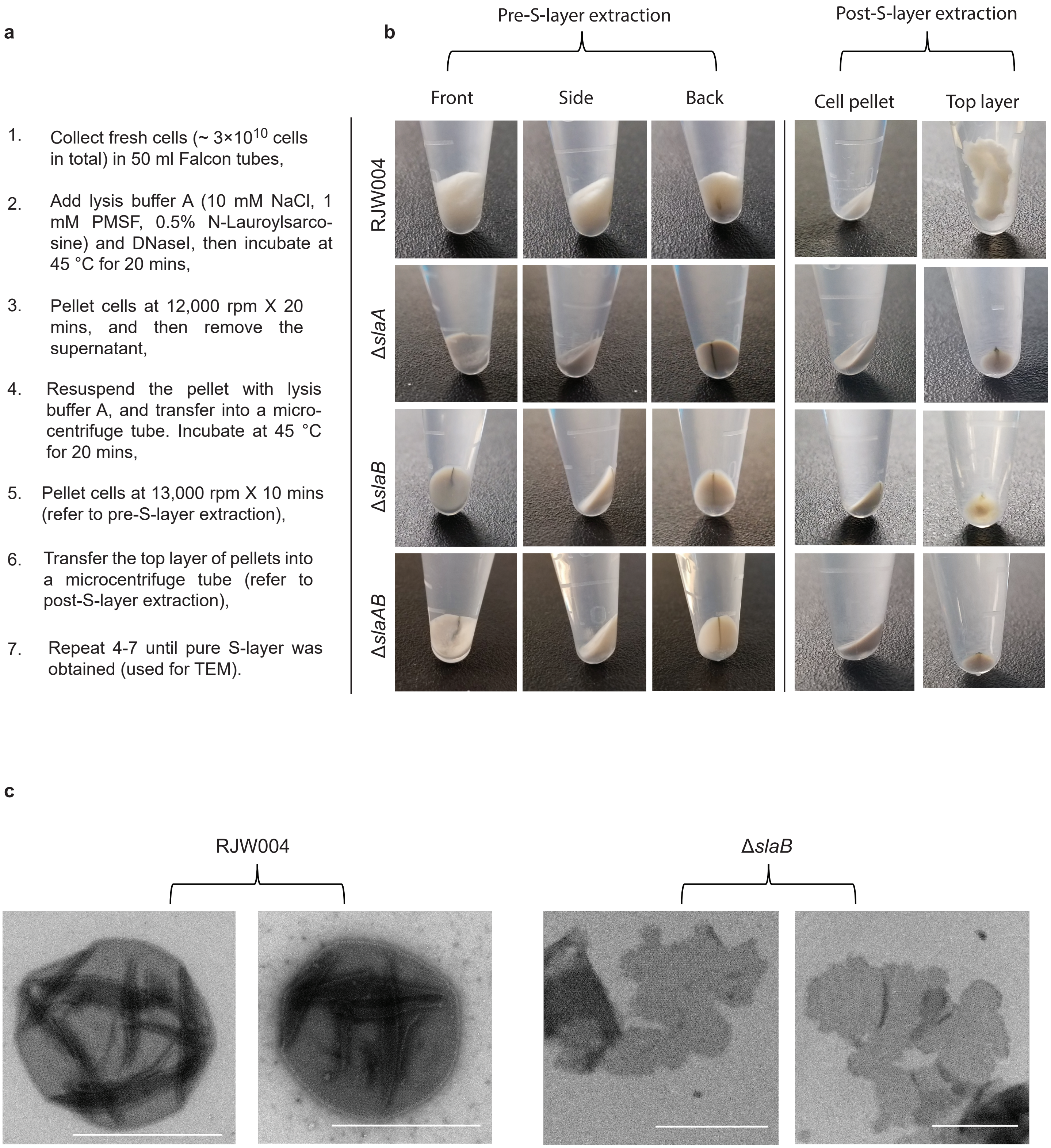

Supplementary Figure 4: Extraction and TEM analysis of S-layer from the wild type (RWJ004) and S-layer gene knockout strains. a, A flowchart illustrating the S-layer extraction procedure, as described previously with minor changes[107]. **b,** Extraction of S-layer from the wild type and S-layer gene knockout strains. A clear whitish layer can be observed and separated from the pellet in the wild type and Δ*slaB* mutant cells, whereas no whitish layer was observed in Δ*slaA* and Δ*slaAB* mutant cells. The whitish layer (S-layer) extracted from the wild type was more abundant than that from the Δ*slaB* mutant cells.These experiments were biologically repeated 3 times and the same phenomenon was observed. **c,** TEM analysis of the negatively stained extracted S-layer (10 µl of MilliQ water-dissolved S-layer) from the wild type and Δ*slaB* mutant cells. Scale bars, 1 µm.

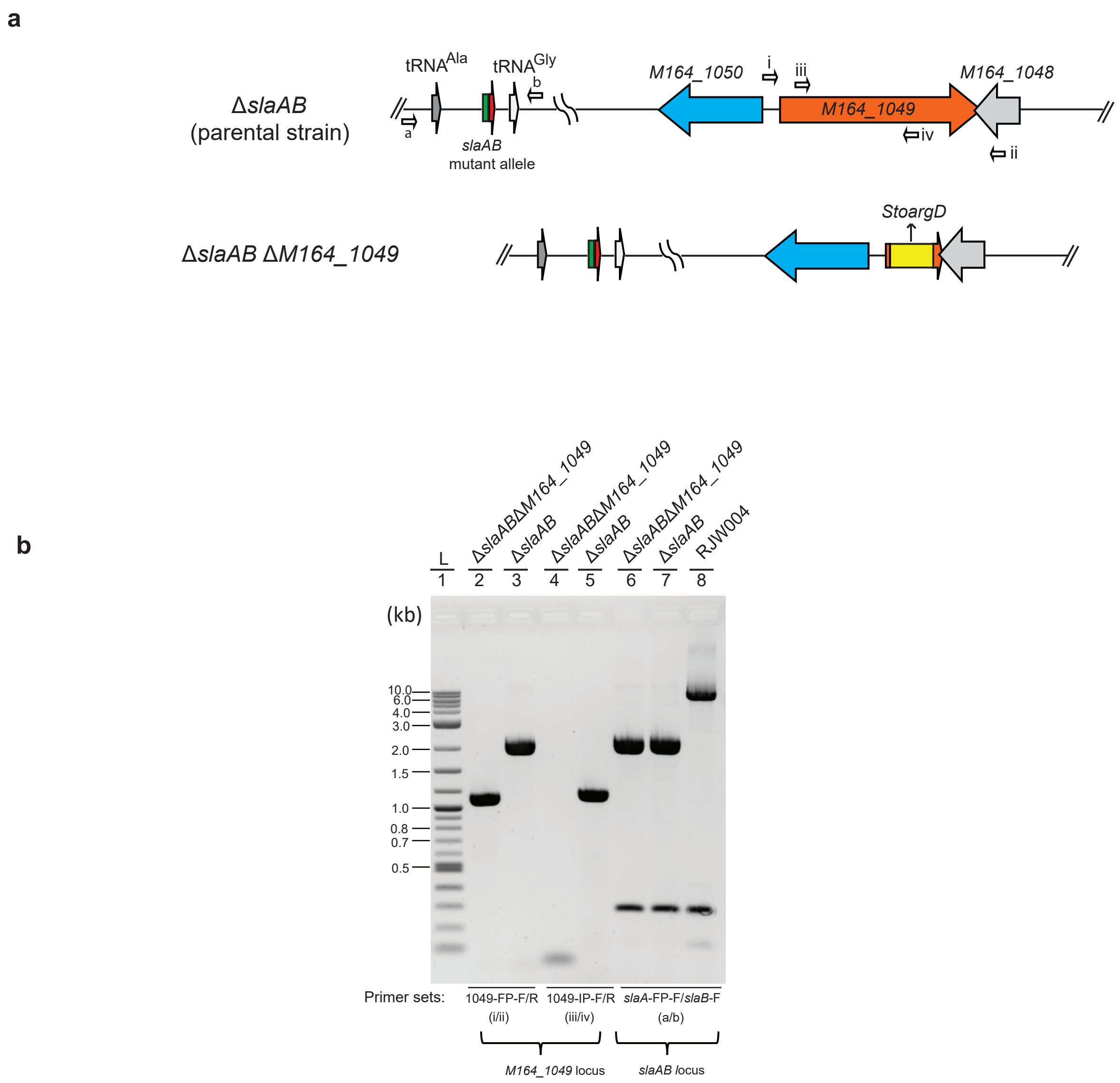

Supplementary Figure 5: Confirmation of the Δ*slaAB*Δ*M164_1049* mutant genotype. a, Genomic context of *M164_1049* in the parental and mutant strains. *M164_1049* was replaced with the selectable marker *StoargD* in the genetic background of the Δ*slaAB* mutant via homologous recombination. **b,** PCR verification of the Δ*slaAB*Δ*M164_1049* mutant strain. The *M164_1049* and *slaAB* loci in the Δ*slaAB*Δ*M164_1049* mutant strain were examined using three primer sets, the relative positions of which are shown with small arrows in **a**. The *S. islandicus* strain RJW004, a genetic host to generate the Δ*slaAB* deletion mutant, is used as a control (lane 8) in PCR analysis when checking the *slaAB* mutant allele in the Δ*slaAB*Δ*M164_1049* mutant strain. L indicates the 2-Log DNA Ladder (NEB, USA), and the marker size corresponding to each band is labelled. Expected sizes of amplicons can be found in Supplementary Table 8.

a

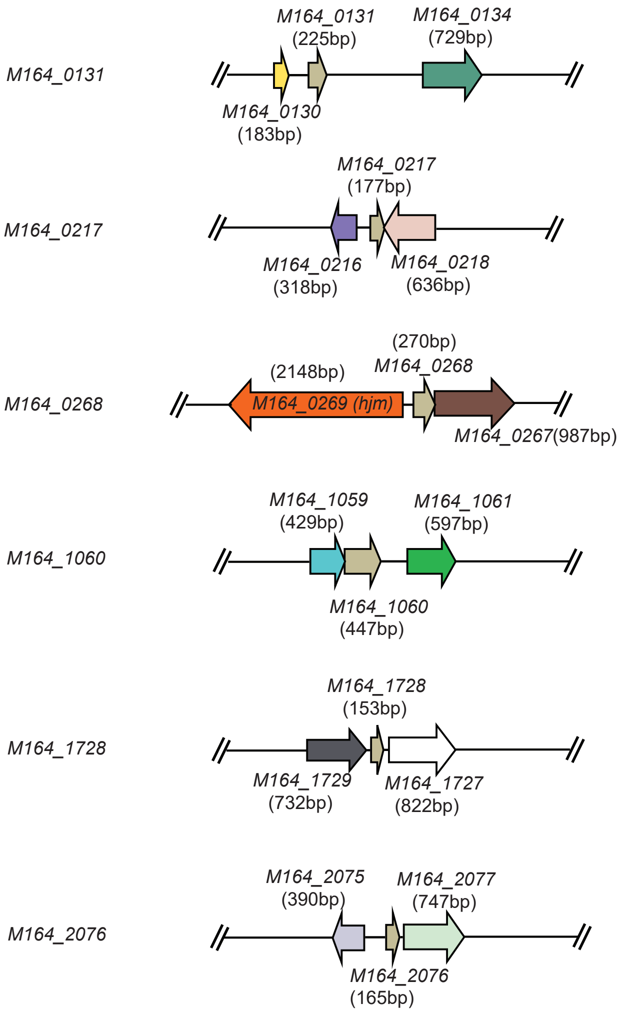

b

Locus_tag	Function	log_2FC	EI	Essential?
M164_0130	Uncharacterized membrane protein	-1.50	8	No
M164_0134	ATPase involved in chromosome partitioning, ParA family	-2.84	9	No
M164_0216	Transcriptional regulator, contains N-terminal RHH domain	1.42	7	No
M164_0218	Zn-dependent hydrolase of the beta-lactamase fold	-2.08	10	No
M164_0267	Phosphate/sulphate permease	0.13	26	No
M164_0269	Replicative superfamily II helicase	-0.89	8	No
M164_1059	HEPN domain containing protein	-1.17	5	No
M164_1061	HAD superfamily hydrolase	1.86	19	No
M164_1727	tRNA(1-methyladenosine) methyltransferase	1.33	20	No
M164_1729	Sugar-specific transcriptional regulator TrmB	-9.84	0	Yes
M164_2075	Transcriptional regulator, contains HTH domain	-8.95	0	Yes
M164_2077	Mg-dependent DNase	-0.13	10	No

Supplementary Figure 6: Predicted essential antitoxin genes. a, Genomic context of the essential antitoxin genes in *S. islandicus* M.16.4. **b,** Essentiality/non-essentiality of genes adjacent to the essential antitoxin genes.

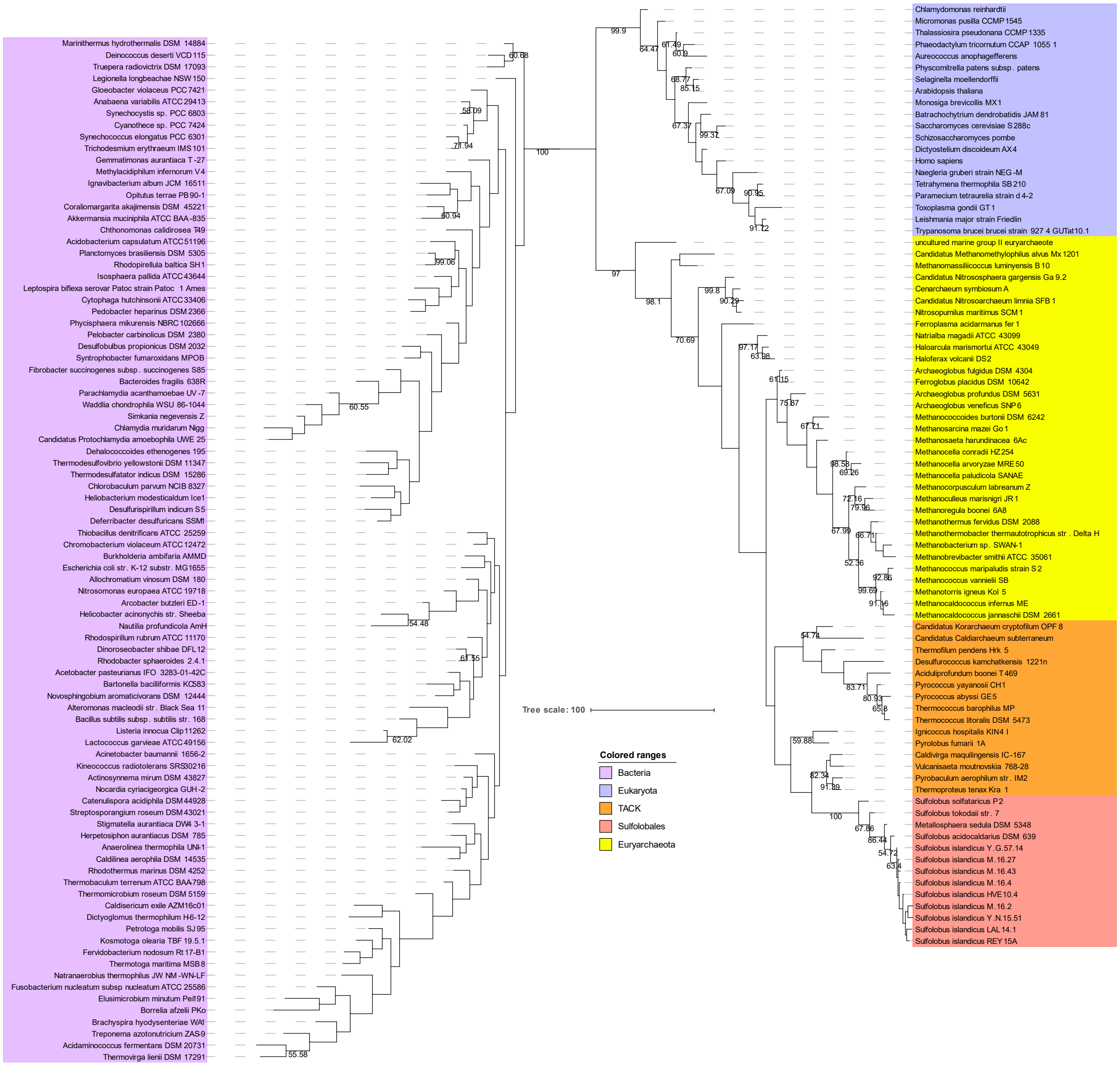

Supplementary Figure 7: Maximum parsimony tree for eggNOG presence/absence patterns. Bootstrap values are shown at nodes where they are greater than 50. Distance is shown in number of changes to the set of genes compared to *S. islandicus* M.16.4.

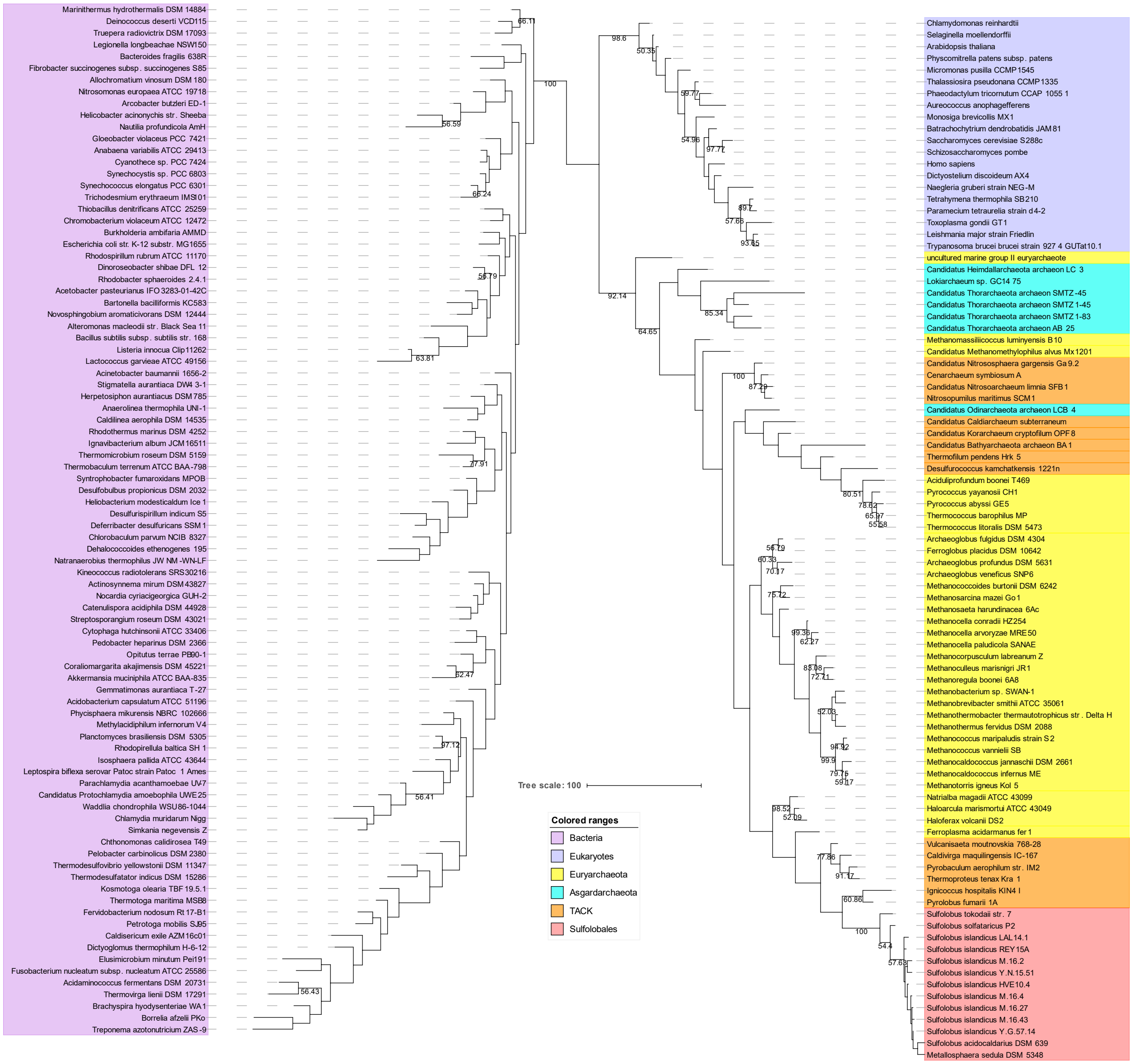

Supplementary Figure 8: Maximum parsimony tree for eggNOG presence/absence patterns, including early Asgardarchaeota genomes. Bootstrap values are shown at nodes where they are greater than 50. Distance is shown in number of changes to the set of genes compared to *S. islandicus* M.16.4.

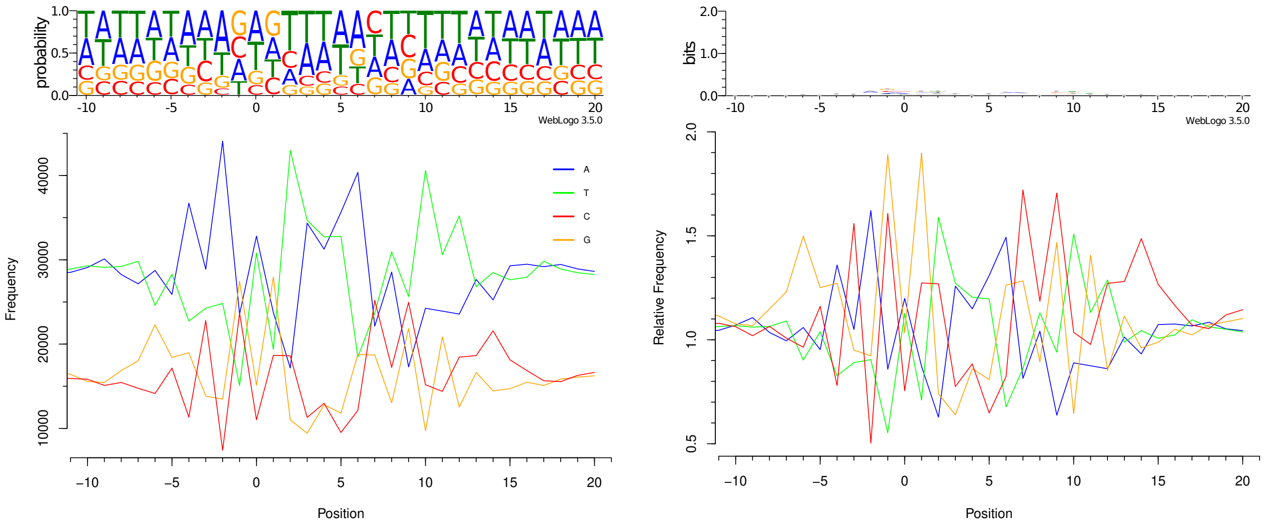

Supplementary Figure 9: Nucleotide frequency near insertion sites shows weak sequence preference compared to random. *Left*, Nucleotide frequency of sequence 10 base-pairs upstream and 20 base-pairs downstream of all insertion sites included in essential gene calculations in logo (top) and line-graph (bottom) forms. *Right*, bits of sequence information in logo form according to WebLogo[108] (top) and frequency relative to counts at an equal number of randomized locations in the genome (bottom).

Supplementary references

1 Sarmiento, F., Mrazek, J. & Whitman, W. B. Genome-scale analysis of gene function in the hydrogenotrophic methanogenic archaeon Methanococcus maripaludis. *Proc Natl Acad Sci U S A* **110**, 4726-4731, doi:10.1073/pnas.1220225110 (2013).

2 Pan, M., Santangelo, T. J., Li, Z., Reeve, J. N. & Kelman, Z. Thermococcus kodakarensis encodes three MCM homologs but only one is essential. *Nucleic Acids Res* **39**, 9671-9680, doi:10.1093/nar/gkr624 (2011).

3 Ishino, S. *et al.* Biochemical and genetical analyses of the three mcm genes from the hyperthermophilic archaeon, Thermococcus kodakarensis. *Genes Cells* **16**, 1176-1189, doi:10.1111/j.1365-2443.2011.01562.x (2011).

4 Liu, W., Pucci, B., Rossi, M., Pisani, F. M. & Ladenstein, R. Structural analysis of the Sulfolobus solfataricus MCM protein N-terminal domain. *Nucleic Acids Res* **36**, 3235-3243, doi:10.1093/nar/gkn183 (2008).

5 Berquist, B. R., DasSarma, P. & DasSarma, S. Essential and non-essential DNA replication genes in the model halophilic Archaeon, Halobacterium sp. NRC-1. *BMC Genet* **8**, 31, doi:10.1186/1471-2156-8-31 (2007).

6 Xu, Y. *et al.* Archaeal orthologs of Cdc45 and GINS form a stable complex that stimulates the helicase activity of MCM. *Proc Natl Acad Sci U S A* **113**, 13390-13395, doi:10.1073/pnas.1613825113 (2016).

7 Burkhart, B. W. *et al.* The GAN Exonuclease or the Flap Endonuclease Fen1 and RNase HII Are Necessary for Viability of Thermococcus kodakarensis. *J Bacteriol* **199**, doi:10.1128/JB.00141-17 (2017).

8 Nagata, M. *et al.* The Cdc45/RecJ-like protein forms a complex with GINS and MCM, and is important for DNA replication in Thermococcus kodakarensis. *Nucleic Acids Res* **45**, 10693-10705, doi:10.1093/nar/gkx740 (2017).

9 Dieckman, L. M., Freudenthal, B. D. & Washington, M. T. PCNA structure and function: insights from structures of PCNA complexes and post-translationally modified PCNA. *Subcell Biochem* **62**, 281-299, doi:10.1007/978-94-007-4572-8_15 (2012).

10 Moldovan, G. L., Pfander, B. & Jentsch, S. PCNA, the maestro of the replication fork. *Cell* **129**, 665-679, doi:10.1016/j.cell.2007.05.003 (2007).

11 Chia, N., Cann, I. & Olsen, G. J. Evolution of DNA replication protein complexes in eukaryotes and Archaea. *PLoS One* **5**, e10866, doi:10.1371/journal.pone.0010866 (2010).

12 Kuba, Y. *et al.* Comparative analyses of the two proliferating cell nuclear antigens from the hyperthermophilic archaeon, Thermococcus kodakarensis. *Genes Cells* **17**, 923-937, doi:10.1111/gtc.12007 (2012).

13 Zhang, C. *et al.* Revealing the essentiality of multiple archaeal pcna genes using a mutant propagation assay based on an improved knockout method. *Microbiology* **156**, 3386-3397, doi:10.1099/mic.0.042523-0 (2010).

14 Dionne, I., Nookala, R. K., Jackson, S. P., Doherty, A. J. & Bell, S. D. A heterotrimeric PCNA in the hyperthermophilic archaeon Sulfolobus solfataricus. *Mol Cell* **11**, 275-282 (2003).

15 Zuo, Z., Rodgers, C. J., Mikheikin, A. L. & Trakselis, M. A. Characterization of a functional DnaG-type primase in archaea: implications for a dual-primase system. *J Mol Biol* **397**, 664-676, doi:10.1016/j.jmb.2010.01.057 (2010).

16 Le Breton, M. *et al.* The heterodimeric primase from the euryarchaeon Pyrococcus abyssi: a multifunctional enzyme for initiation and repair? *J Mol Biol* **374**, 1172-1185, doi:10.1016/j.jmb.2007.10.015 (2007).

17 Liu, B. *et al.* A primase subunit essential for efficient primer synthesis by an archaeal eukaryotic-type primase. *Nat Commun* **6**, 7300, doi:10.1038/ncomms8300 (2015).

18 Lai, X., Shao, H., Hao, F. & Huang, L. Biochemical characterization of an ATP-dependent DNA ligase from the hyperthermophilic crenarchaeon Sulfolobus shibatae. *Extremophiles* **6**, 469-477, doi:10.1007/s00792-002-0284-5 (2002).

19 Zhan, K. & He, Z. G. Characterization of a new RNase HII and its essential amino acid residues in the archaeon Sulfolobus tokodaii reveals a regulatory C-terminus. *Biochemistry (Mosc)* **75**, 930-937 (2010).
20 Choi, J. Y. *et al.* Roles of the four DNA polymerases of the crenarchaeon Sulfolobus solfataricus and accessory proteins in DNA replication. *J Biol Chem* **286**, 31180-31193, doi:10.1074/jbc.M111.258038 (2011).
21 Taylor, K. A., Deatherage, J. F. & Amos, L. A. Structure of the S-Layer of Sulfolobus-Acidocaldarius. *Nature* **299**, 840-842, doi:DOI 10.1038/299840a0 (1982).
22 Edgell, D. R., Klenk, H. P. & Doolittle, W. F. Gene duplications in evolution of archaeal family B DNA polymerases. *J Bacteriol* **179**, 2632-2640 (1997).
23 Edgell, D. R., Malik, S. B. & Doolittle, W. F. Evidence of independent gene duplications during the evolution of archaeal and eukaryotic family B DNA polymerases. *Mol Biol Evol* **15**, 1207-1217, doi:10.1093/oxfordjournals.molbev.a026028 (1998).
24 Yan, J. *et al.* Identification and characterization of a heterotrimeric archaeal DNA polymerase holoenzyme. *Nat Commun* **8**, 15075, doi:10.1038/ncomms15075 (2017).
25 Abby, S. S. *et al.* Candidatus Nitrosocaldus cavascurensis, an Ammonia Oxidizing, Extremely Thermophilic Archaeon with a Highly Mobile Genome. *Front Microbiol* **9**, 28, doi:10.3389/fmicb.2018.00028 (2018).
26 Daebeler, A. *et al.* Cultivation and Genomic Analysis of "Candidatus Nitrosocaldus islandicus," an Obligately Thermophilic, Ammonia-Oxidizing Thaumarchaeon from a Hot Spring Biofilm in Graendalur Valley, Iceland. *Front Microbiol* **9**, 193, doi:10.3389/fmicb.2018.00193 (2018).
27 Cubonova, L. *et al.* Archaeal DNA polymerase D but not DNA polymerase B is required for genome replication in Thermococcus kodakarensis. *J Bacteriol* **195**, 2322-2328, doi:10.1128/JB.02037-12 (2013).
28 Adam, P. S., Borrel, G., Brochier-Armanet, C. & Gribaldo, S. The growing tree of Archaea: new perspectives on their diversity, evolution and ecology. *ISME J* **11**, 2407-2425, doi:10.1038/ismej.2017.122 (2017).
29 Guo, L. *et al.* Biochemical and structural characterization of Cren7, a novel chromatin protein conserved among Crenarchaea. *Nucleic Acids Res* **36**, 1129-1137, doi:10.1093/nar/gkm1128 (2008).
30 Ishino, S. *et al.* Identification of a mismatch-specific endonuclease in hyperthermophilic Archaea. *Nucleic Acids Res* **44**, 2977-2986, doi:10.1093/nar/gkw153 (2016).
31 White, M. F. & Allers, T. DNA Repair in the Archaea - an emerging picture. *FEMS Microbiol Rev*, doi:10.1093/femsre/fuy020 (2018).
32 Castaneda-Garcia, A. *et al.* A non-canonical mismatch repair pathway in prokaryotes. *Nat Commun* **8**, 14246, doi:10.1038/ncomms14246 (2017).
33 Ishino, S. *et al.* Activation of the mismatch-specific endonuclease EndoMS/NucS by the replication clamp is required for high fidelity DNA replication. *Nucleic Acids Res*, doi:10.1093/nar/gky460 (2018).
34 Zhang, C. *et al.* Genetic manipulation in Sulfolobus islandicus and functional analysis of DNA repair genes. *Biochem Soc Trans* **41**, 405-410, doi:10.1042/BST20120285 (2013).
35 Fujikane, R., Ishino, S., Ishino, Y. & Forterre, P. Genetic analysis of DNA repair in the hyperthermophilic archaeon, Thermococcus kodakaraensis. *Genes Genet Syst* **85**, 243-257 (2010).
36 Woods, W. G. & Dyall-Smith, M. L. Construction and analysis of a recombination-deficient (radA) mutant of Haloferax volcanii. *Mol Microbiol* **23**, 791-797 (1997).
37 Hawkins, M., Malla, S., Blythe, M. J., Nieduszynski, C. A. & Allers, T. Accelerated growth in the absence of DNA replication origins. *Nature* **503**, 544-547, doi:10.1038/nature12650 (2013).

38 Delmas, S., Duggin, I. G. & Allers, T. DNA damage induces nucleoid compaction via the Mre11-Rad50 complex in the archaeon Haloferax volcanii. *Mol Microbiol* **87**, 168-179, doi:10.1111/mmi.12091 (2013).

39 Kish, A. & DiRuggiero, J. Rad50 is not essential for the Mre11-dependent repair of DNA double-strand breaks in Halobacterium sp. strain NRC-1. *J Bacteriol* **190**, 5210-5216, doi:10.1128/JB.00292-08 (2008).

40 Delmas, S., Shunburne, L., Ngo, H. P. & Allers, T. Mre11-Rad50 promotes rapid repair of DNA damage in the polyploid archaeon Haloferax volcanii by restraining homologous recombination. *PLoS Genet* **5**, e1000552, doi:10.1371/journal.pgen.1000552 (2009).

41 Grogan, D. W. Understanding DNA Repair in Hyperthermophilic Archaea: Persistent Gaps and Other Reasons to Focus on the Fork. *Archaea* **2015**, 942605, doi:10.1155/2015/942605 (2015).

42 Korkhin, Y. *et al.* Evolution of complex RNA polymerases: the complete archaeal RNA polymerase structure. *PLoS Biol* **7**, e1000102, doi:10.1371/journal.pbio.1000102 (2009).

43 Koonin, E. V., Makarova, K. S. & Elkins, J. G. Orthologs of the small RPB8 subunit of the eukaryotic RNA polymerases are conserved in hyperthermophilic Crenarchaeota and "Korarchaeota". *Biol Direct* **2**, 38, doi:10.1186/1745-6150-2-38 (2007).

44 Wojtas, M. N., Mogni, M., Millet, O., Bell, S. D. & Abrescia, N. G. Structural and functional analyses of the interaction of archaeal RNA polymerase with DNA. *Nucleic Acids Res* **40**, 9941-9952, doi:10.1093/nar/gks692 (2012).

45 Iqbal, J. & Qureshi, S. A. Selective depletion of Sulfolobus solfataricus transcription factor E under heat shock conditions. *J Bacteriol* **192**, 2887-2891, doi:10.1128/JB.01534-09 (2010).

46 Rauch, B. Functional analysis of multiple general transcription factors in Sulfolobus acidocaldarius. *PhD Thesis* (2013).

47 Qureshi, S. A., Bell, S. D. & Jackson, S. P. Factor requirements for transcription in the Archaeon Sulfolobus shibatae. *EMBO J* **16**, 2927-2936, doi:10.1093/emboj/16.10.2927 (1997).

48 Lundgren, M. & Bernander, R. Genome-wide transcription map of an archaeal cell cycle. *Proc Natl Acad Sci U S A* **104**, 2939-2944, doi:10.1073/pnas.0611333104 (2007).

49 Gotz, D. *et al.* Responses of hyperthermophilic crenarchaea to UV irradiation. *Genome Biol* **8**, R220, doi:10.1186/gb-2007-8-10-r220 (2007).

50 Frols, S. *et al.* Response of the hyperthermophilic archaeon Sulfolobus solfataricus to UV damage. *J Bacteriol* **189**, 8708-8718, doi:10.1128/JB.01016-07 (2007).

51 Feng, X., Sun, M., Han, W., Liang, Y. X. & She, Q. A transcriptional factor B paralog functions as an activator to DNA damage-responsive expression in archaea. *Nucleic Acids Res*, doi:10.1093/nar/gky236 (2018).

52 Schult, F. *et al.* Effect of UV irradiation on Sulfolobus acidocaldarius and involvement of the general transcription factor TFB3 in the early UV response. *Nucleic Acids Res*, doi:10.1093/nar/gky527 (2018).

53 Blombach, F. *et al.* Archaeal TFEalpha/beta is a hybrid of TFIIE and the RNA polymerase III subcomplex hRPC62/39. *Elife* **4**, e08378, doi:10.7554/eLife.08378 (2015).

54 Daniels, J. P., Kelly, S., Wickstead, B. & Gull, K. Identification of a crenarchaeal orthologue of Elf1: implications for chromatin and transcription in Archaea. *Biol Direct* **4**, 24, doi:10.1186/1745-6150-4-24 (2009).

55 Fouqueau, T. *et al.* The transcript cleavage factor paralogue TFS4 is a potent RNA polymerase inhibitor. *Nat Commun* **8**, 1914, doi:10.1038/s41467-017-02081-3 (2017).

56 Fischer, S. *et al.* The archaeal Lsm protein binds to small RNAs. *J Biol Chem* **285**, 34429-34438, doi:10.1074/jbc.M110.118950 (2010).

57 Martens, B. *et al.* The Heptameric SmAP1 and SmAP2 Proteins of the Crenarchaeon Sulfolobus Solfataricus Bind to Common and Distinct RNA Targets. *Life (Basel)* **5**, 1264-1281, doi:10.3390/life5021264 (2015).
58 Martens, B. *et al.* The SmAP1/2 proteins of the crenarchaeon Sulfolobus solfataricus interact with the exosome and stimulate A-rich tailing of transcripts. *Nucleic Acids Res* **45**, 7938-7949, doi:10.1093/nar/gkx437 (2017).
59 Gangwani, L., Mikrut, M., Theroux, S., Sharma, M. & Davis, R. J. Spinal muscular atrophy disrupts the interaction of ZPR1 with the SMN protein. *Nat Cell Biol* **3**, 376-383, doi:10.1038/35070059 (2001).
60 Gangwani, L., Flavell, R. A. & Davis, R. J. ZPR1 is essential for survival and is required for localization of the survival motor neurons (SMN) protein to Cajal bodies. *Mol Cell Biol* **25**, 2744-2756, doi:10.1128/MCB.25.7.2744-2756.2005 (2005).
61 Gangwani, L. Deficiency of the zinc finger protein ZPR1 causes defects in transcription and cell cycle progression. *J Biol Chem* **281**, 40330-40340, doi:10.1074/jbc.M608165200 (2006).
62 Ahel, I., Korencic, D., Ibba, M. & Soll, D. Trans-editing of mischarged tRNAs. *Proc Natl Acad Sci U S A* **100**, 15422-15427, doi:10.1073/pnas.2136934100 (2003).
63 Tumbula, D. *et al.* Archaeal aminoacyl-tRNA synthesis: diversity replaces dogma. *Genetics* **152**, 1269-1276 (1999).
64 Tumbula, D. L., Becker, H. D., Chang, W. Z. & Soll, D. Domain-specific recruitment of amide amino acids for protein synthesis. *Nature* **407**, 106-110, doi:10.1038/35024120 (2000).
65 Sheppard, K. & Soll, D. On the evolution of the tRNA-dependent amidotransferases, GatCAB and GatDE. *J Mol Biol* **377**, 831-844, doi:10.1016/j.jmb.2008.01.016 (2008).
66 Atkinson, G. C., Hauryliuk, V. & Tenson, T. An ancient family of SelB elongation factor-like proteins with a broad but disjunct distribution across archaea. *BMC Evol Biol* **11**, 22, doi:10.1186/1471-2148-11-22 (2011).
67 Lindas, A. C., Karlsson, E. A., Lindgren, M. T., Ettema, T. J. & Bernander, R. A unique cell division machinery in the Archaea. *Proc Natl Acad Sci U S A* **105**, 18942-18946, doi:10.1073/pnas.0809467105 (2008).
68 Samson, R. Y., Obita, T., Freund, S. M., Williams, R. L. & Bell, S. D. A role for the ESCRT system in cell division in archaea. *Science* **322**, 1710-1713, doi:10.1126/science.1165322 (2008).
69 Yang, N. & Driessen, A. J. Deletion of cdvB paralogous genes of Sulfolobus acidocaldarius impairs cell division. *Extremophiles* **18**, 331-339, doi:10.1007/s00792-013-0618-5 (2014).
70 Liu, J. *et al.* Functional assignment of multiple ESCRT-III homologs in cell division and budding in Sulfolobus islandicus. *Mol Microbiol* **105**, 540-553, doi:10.1111/mmi.13716 (2017).
71 Zhang, C. & Whitaker, R. J. Microhomology-Mediated High-Throughput Gene Inactivation Strategy for the Hyperthermophilic Crenarchaeon Sulfolobus islandicus. *Appl Environ Microbiol* **84**, doi:10.1128/AEM.02167-17 (2018).
72 Kalliomaa-Sanford, A. K. *et al.* Chromosome segregation in Archaea mediated by a hybrid DNA partition machine. *Proc Natl Acad Sci U S A* **109**, 3754-3759, doi:10.1073/pnas.1113384109 (2012).
73 Snijders, A. P. *et al.* Reconstruction of central carbon metabolism in Sulfolobus solfataricus using a two-dimensional gel electrophoresis map, stable isotope labelling and DNA microarray analysis. *Proteomics* **6**, 1518-1529, doi:10.1002/pmic.200402070 (2006).
74 Ulas, T., Riemer, S. A., Zaparty, M., Siebers, B. & Schomburg, D. Genome-scale reconstruction and analysis of the metabolic network in the hyperthermophilic archaeon Sulfolobus solfataricus. *PLoS One* **7**, e43401, doi:10.1371/journal.pone.0043401 (2012).

75 Yan, Z., Maruyama, A., Arakawa, T., Fushinobu, S. & Wakagi, T. Crystal structures of archaeal 2-oxoacid:ferredoxin oxidoreductases from Sulfolobus tokodaii. *Sci Rep* **6**, 33061, doi:10.1038/srep33061 (2016).
76 Samson, R. Y. *et al.* Specificity and function of archaeal DNA replication initiator proteins. *Cell Rep* **3**, 485-496, doi:10.1016/j.celrep.2013.01.002 (2013).
77 Liang, P. J. *et al.* Knockouts of RecA-like proteins RadC1 and RadC2 have distinct responses to DNA damage agents in Sulfolobus islandicus. *J Genet Genomics* **40**, 533-542, doi:10.1016/j.jgg.2013.05.004 (2013).
78 Huang, Q. *et al.* Genetic analysis of the Holliday junction resolvases Hje and Hjc in Sulfolobus islandicus. *Extremophiles* **19**, 505-514, doi:10.1007/s00792-015-0734-5 (2015).
79 Chu, Y. *et al.* aKMT Catalyzes Extensive Protein Lysine Methylation in the Hyperthermophilic Archaeon Sulfolobus islandicus but is Dispensable for the Growth of the Organism. *Mol Cell Proteomics* **15**, 2908-2923, doi:10.1074/mcp.M115.057778 (2016).
80 Li, X. *et al.* Deletion of the topoisomerase III gene in the hyperthermophilic archaeon Sulfolobus islandicus results in slow growth and defects in cell cycle control. *J Genet Genomics* **38**, 253-259, doi:10.1016/j.jgg.2011.05.001 (2011).
81 Zhang, C., Cooper, T. E., Krause, D. J. & Whitaker, R. J. Augmenting the genetic toolbox for Sulfolobus islandicus with a stringent positive selectable marker for agmatine prototrophy. *Appl Environ Microbiol* **79**, 5539-5549, doi:10.1128/AEM.01608-13 (2013).
82 Zhang, C. & Whitaker, R. J. A broadly applicable gene knockout system for the thermoacidophilic archaeon Sulfolobus islandicus based on simvastatin selection. *Microbiology* **158**, 1513-1522, doi:10.1099/mic.0.058289-0 (2012).
83 Zhang, C., She, Q., Bi, H. & Whitaker, R. J. The apt/6-Methylpurine Counterselection System and Its Applications in Genetic Studies of the Hyperthermophilic Archaeon Sulfolobus islandicus. *Appl Environ Microbiol* **82**, 3070-3081, doi:10.1128/AEM.00455-16 (2016).
84 Bautista, M. A., Zhang, C. & Whitaker, R. J. Virus-induced dormancy in the archaeon Sulfolobus islandicus. *MBio* **6**, doi:10.1128/mBio.02565-14 (2015).
85 Peng, W. *et al.* Genetic determinants of PAM-dependent DNA targeting and pre-crRNA processing in Sulfolobus islandicus. *RNA Biol* **10**, 738-748, doi:10.4161/rna.23798 (2013).
86 Li, Y. *et al.* Harnessing Type I and Type III CRISPR-Cas systems for genome editing. *Nucleic Acids Res* **44**, e34, doi:10.1093/nar/gkv1044 (2016).
87 Liu, T. *et al.* Transcriptional regulator-mediated activation of adaptation genes triggers CRISPR de novo spacer acquisition. *Nucleic Acids Res* **43**, 1044-1055, doi:10.1093/nar/gku1383 (2015).
88 He, F., Vestergaard, G., Peng, W., She, Q. & Peng, X. CRISPR-Cas type I-A Cascade complex couples viral infection surveillance to host transcriptional regulation in the dependence of Csa3b. *Nucleic Acids Res* **45**, 1902-1913, doi:10.1093/nar/gkw1265 (2017).
89 Deng, L., Kenchappa, C. S., Peng, X., She, Q. & Garrett, R. A. Modulation of CRISPR locus transcription by the repeat-binding protein Cbp1 in Sulfolobus. *Nucleic Acids Res* **40**, 2470-2480, doi:10.1093/nar/gkr1111 (2012).
90 Zhai, B. *et al.* Structure and Function of a Novel ATPase that Interacts with Holliday Junction Resolvase Hjc and Promotes Branch Migration. *J Mol Biol* **429**, 1009-1029, doi:10.1016/j.jmb.2017.02.016 (2017).
91 Song, X., Huang, Q., Ni, J., Yu, Y. & Shen, Y. Knockout and functional analysis of two DExD/H-box family helicase genes in Sulfolobus islandicus REY15A. *Extremophiles* **20**, 537-546, doi:10.1007/s00792-016-0847-5 (2016).
92 Puigbo, P., Wolf, Y. I. & Koonin, E. V. Search for a 'Tree of Life' in the thicket of the phylogenetic forest. *J Biol* **8**, 59, doi:10.1186/jbiol159 (2009).

93 Harris, J. K., Kelley, S. T., Spiegelman, G. B. & Pace, N. R. The genetic core of the universal ancestor. *Genome Res* **13**, 407-412, doi:10.1101/gr.652803 (2003).
94 Gil, R. *et al.* The genome sequence of Blochmannia floridanus: comparative analysis of reduced genomes. *Proc Natl Acad Sci U S A* **100**, 9388-9393, doi:10.1073/pnas.1533499100 (2003).
95 Weiss, M. C. *et al.* The physiology and habitat of the last universal common ancestor. *Nat Microbiol* **1**, 16116, doi:10.1038/nmicrobiol.2016.116 (2016).
96 Wolf, Y. I., Makarova, K. S., Yutin, N. & Koonin, E. V. Updated clusters of orthologous genes for Archaea: a complex ancestor of the Archaea and the byways of horizontal gene transfer. *Biol Direct* **7**, 46, doi:10.1186/1745-6150-7-46 (2012).
97 Guy, L. & Ettema, T. J. The archaeal 'TACK' superphylum and the origin of eukaryotes. *Trends Microbiol* **19**, 580-587, doi:10.1016/j.tim.2011.09.002 (2011).
98 Raymann, K., Brochier-Armanet, C. & Gribaldo, S. The two-domain tree of life is linked to a new root for the Archaea. *Proc Natl Acad Sci U S A* **112**, 6670-6675, doi:10.1073/pnas.1420858112 (2015).
99 Eme, L., Spang, A., Lombard, J., Stairs, C. W. & Ettema, T. J. G. Archaea and the origin of eukaryotes. *Nat Rev Microbiol* **15**, 711-723, doi:10.1038/nrmicro.2017.133 (2017).
100 Makarova, K. S., Yutin, N., Bell, S. D. & Koonin, E. V. Evolution of diverse cell division and vesicle formation systems in Archaea. *Nat Rev Microbiol* **8**, 731-741, doi:10.1038/nrmicro2406 (2010).
101 Yutin, N., Puigbo, P., Koonin, E. V. & Wolf, Y. I. Phylogenomics of prokaryotic ribosomal proteins. *PLoS One* **7**, e36972, doi:10.1371/journal.pone.0036972 (2012).
102 Mirkin, B. G., Fenner, T. I., Galperin, M. Y. & Koonin, E. V. Algorithms for computing parsimonious evolutionary scenarios for genome evolution, the last universal common ancestor and dominance of horizontal gene transfer in the evolution of prokaryotes. *BMC Evol Biol* **3**, 2 (2003).
103 Makarova, K. S., Wolf, Y. I. & Koonin, E. V. Archaeal Clusters of Orthologous Genes (arCOGs): An Update and Application for Analysis of Shared Features between Thermococcales, Methanococcales, and Methanobacteriales. *Life (Basel)* **5**, 818-840, doi:10.3390/life5010818 (2015).
104 Reno, M. L., Held, N. L., Fields, C. J., Burke, P. V. & Whitaker, R. J. Biogeography of the Sulfolobus islandicus pan-genome. *Proc Natl Acad Sci U S A* **106**, 8605-8610, doi:10.1073/pnas.0808945106 (2009).
105 Zhang, C. & Whitaker, R. J. Microhomology Mediated High-throughput Gene Inactivation Strategy for Hyperthermophilic Crenarchaeon Sulfolobus islandicus. *Appl Environ Microbiol*, doi:10.1128/AEM.02167-17 (2017).
106 Peng, N. *et al.* A synthetic arabinose-inducible promoter confers high levels of recombinant protein expression in hyperthermophilic archaeon Sulfolobus islandicus. *Appl Environ Microbiol* **78**, 5630-5637, doi:10.1128/AEM.00855-12 (2012).
107 Peyfoon, E. *et al.* The S-layer glycoprotein of the crenarchaeote Sulfolobus acidocaldarius is glycosylated at multiple sites with chitobiose-linked N-glycans. *Archaea* **2010**, doi:10.1155/2010/754101 (2010).
108 Crooks, G. E., Hon, G., Chandonia, J. M. & Brenner, S. E. WebLogo: a sequence logo generator. *Genome Res* **14**, 1188-1190, doi:10.1101/gr.849004 (2004).
