# Supplementary material for: The essential genome of the crenarchaeal model Sulfolobus islandicus
Source: Nat Commun. 2018 Nov 21;9:4908. doi: 10.1038/s41467-018-07379-4 (PMC6249222; doi:10.1038/s41467-018-07379-4)
Supplement: Supplementary file 2 — Description of Additional Supplementary Files [file 41467_2018_7379_MOESM2_ESM.pdf]

Description of Additional Supplementary Files

File Name: Supplementary Data 1

Description: Reads, orientation, and start position for each transposon insertion in each library. Numbers were extracted from a .sam file that was generated as specified in Methods.

File Name: Supplementary Data 2

Description: Gene information/annotation for *S. islandicus* M.16.4, including essentiality assignments. Putative orthologs in other Sulfolobales are also included and multiple tabs are provided for different gene sets.

File Name: Supplementary Data 3

Description: Predicted toxin-antitoxin pairs in the genome of *S. islandicus* M.16.4. Two types of putative toxin-antitoxin (TA) pairs are found in the *S. islandicus* M.16.4 genome including: 1) 22 of VapBC (Virulence Associated Proteins B and C) TA pairs, and 2) 8 of HEPN-NT (Higher Eukaryote and Prokaryote Nucleotide binding-Nucleotidylransferases) TA pairs.

File Name: Supplementary Data 4

Description: Presence, absence, and essentiality information for each *S. islandicus* M.16.4 essential gene compared to selected previous essential genome projects. Generated as described in Methods, these data are the basis for Figure 5.

File Name: Supplementary Data 5

Description: Presence/absence assignments of each essential gene across selected genomes. These data are the basis for Figure 6. Genomes were selected as described in Methods.

File Name: Supplementary Data 6

Description: Strain names for each genome in Supplementary Data 5 with associated NCBI taxonomy. These categorizations were the basis for phyletic categories in Tables 1 and 2, as well as Supplementary Data 7. Genomes were selected as described in Methods.

File Name: Supplementary Data 7

Description: Phyletic category assignments for each essential gene based on presence/absence data. This is based on the cutoffs specified in Tables 1 and 2, and are based on the data in Supplementary Data 5.

File Name: Supplementary Data 8

Description: Presence/absence of essential genes across selected previous gene ortholog distribution data sets. The matching gene, protein, or orthologous group identifier is indicated for each gene for each data set, and citations can be found in Supplementary Table 5.

File Name: Supplementary Data 9

Description: Primers used in this study. Primers in this study are used for three different purposes including: 1) transposon mutagenesis, 2) gene knockout analysis, and 3) verification of gene deletion.

File Name: Supplementary Data 10

Description: Annotations of genes that were performed genetic analysis in *S. islandicus*. The genes that have knockout analyses in previous or current study are surveyed in three *S. islandicus* strains REY15A, LAL1/14, and M.16.4. The essentiality/non-essentiality of these genes are shown by both Tn-seq and knockout analyses, as seen in Supplementary Table 3.
