# Supplementary material for: The essential genome of the crenarchaeal model Sulfolobus islandicus
Source: Nat Commun. 2018 Nov 21;9:4908. doi: 10.1038/s41467-018-07379-4 (PMC6249222; doi:10.1038/s41467-018-07379-4)
Supplement: Supplementary file 13 — Reporting Summary [file 41467_2018_7379_MOESM13_ESM.pdf]

nature research

Corresponding author(s): Rachel J. Whitaker

Reporting Summary

Nature Research wishes to improve the reproducibility of the work that we publish. This form provides structure for consistency and transparency in reporting. For further information on Nature Research policies, see Authors & Referees and the Editorial Policy Checklist.

Statistical parameters

When statistical analyses are reported, confirm that the following items are present in the relevant location (e.g. figure legend, table legend, main text, or Methods section).

n/a	Confirmed	
☐	☒	The exact sample size (*n*) for each experimental group/condition, given as a discrete number and unit of measurement
☐	☒	An indication of whether measurements were taken from distinct samples or whether the same sample was measured repeatedly
☐	☒	The statistical test(s) used AND whether they are one- or two-sided<br>*Only common tests should be described solely by name; describe more complex techniques in the Methods section.*
☐	☒	A description of all covariates tested
☐	☒	A description of any assumptions or corrections, such as tests of normality and adjustment for multiple comparisons
☐	☒	A full description of the statistics including central tendency (e.g. means) or other basic estimates (e.g. regression coefficient) AND variation (e.g. standard deviation) or associated estimates of uncertainty (e.g. confidence intervals)
☐	☒	For null hypothesis testing, the test statistic (e.g. *F*, *t*, *r*) with confidence intervals, effect sizes, degrees of freedom and *P* value noted<br>*Give P values as exact values whenever suitable.*
☐	☒	For Bayesian analysis, information on the choice of priors and Markov chain Monte Carlo settings
☐	☒	For hierarchical and complex designs, identification of the appropriate level for tests and full reporting of outcomes
☐	☒	Estimates of effect sizes (e.g. Cohen's *d*, Pearson's *r*), indicating how they were calculated
☐	☒	Clearly defined error bars<br>*State explicitly what error bars represent (e.g. SD, SE, CI)*

Our web collection on statistics for biologists may be useful.

Software and code

Policy information about availability of computer code

Data collection	The 169 genome sequences used in this study are retrieved from original publications or NCBI.
Data analysis	Custom python scripts were used for processing transposon insertion sequencing data, as well as for organizing data, quantifying taxonomic distribution, and comparing observed distributions to random. Two computational approaches i.e. Tn-Seq Explorer and ESSENTIALS were used to evaluate gene essentiality. Two programs, HMMER and eggNOG mapper, were used for sequence search. PAUP* was used to construct maximum parsimony trees. Code is available upon request. ITOL was used to visualize trees.

For manuscripts utilizing custom algorithms or software that are central to the research but not yet described in published literature, software must be made available to editors/reviewers upon request. We strongly encourage code deposition in a community repository (e.g. GitHub). See the Nature Research guidelines for submitting code & software for further information.

Data

Policy information about availability of data

All manuscripts must include a data availability statement. This statement should provide the following information, where applicable:

- Accession codes, unique identifiers, or web links for publicly available datasets
- A list of figures that have associated raw data
- A description of any restrictions on data availability

The raw Tn-seq data of three independent transposon insertion libraries CYZ-TL1, CYZ-TL2, and CYZ-TL3 have been deposited at NCBI under BioSample accessions SAMN08628694, SAMN08628695, SAMN08628696, respectively, Bioproject accession PRJNA436600, and Sequence Read Archive (SRA) accession SRP133799. Analyzed data showing the insertion locations across three independent transposon libraries can be found in Supplementary Dataset 1. All other data that support the findings of this work are available from the corresponding author upon request.

Field-specific reporting

Please select the best fit for your research. If you are not sure, read the appropriate sections before making your selection.

☒ Life sciences ☐ Behavioural & social sciences ☐ Ecological, evolutionary & environmental sciences

For a reference copy of the document with all sections, see nature.com/authors/policies/ReportingSummary-flat.pdf

Life sciences study design

All studies must disclose on these points even when the disclosure is negative.

Sample size	The theoretical number of transposon insertion colonies was calculated using a derivative of Poisson's law: N = ln(1 - P)/ln(1 - f), f = average gene size (900.64 bp) / genome size (2,586,647bp). To make sure the transposon insertions cover approximately 99.99% (P=0.9999) of the genome, around 26,448 colonies per library are required.
Data exclusions	No data was excluded.
Replication	Experimental findings were reliably reproducible, and were replicated as described in the paper.
Randomization	Three independent transposon insertion mutant libraries were made in this study. The number of mutants in each mutant library varies, but it is sufficient to make sure that 99.99% of genome can be covered by transposon insertions. Randomization of genomic locations in analyzing insertion site bias was carried out with the Python package numpy.random.choice, with replacement. Randomization of gene distributions were carried out using the Python numpy.random.choice package, using the original number of organisms in which a gene was found to generate a random set of organisms without replacement. This same method was used to sub-sample organisms from the eggNOG database as described in methods.
Blinding	Blinding was not used for the data collection and analysis.

Reporting for specific materials, systems and methods

Materials & experimental systems

n/a	Involved in the study
☒	☐ Unique biological materials
☒	☐ Antibodies
☒	☐ Eukaryotic cell lines
☒	☐ Palaeontology
☒	☐ Animals and other organisms
☒	☐ Human research participants

Methods

n/a	Involved in the study
☒	☐ ChIP-seq
☒	☐ Flow cytometry
☒	☐ MRI-based neuroimaging
